# Rapid aqueous-phase dark reaction of phenols with nitrosonium ions: Novel mechanism for atmospheric nitrosation and nitration at low pH

**DOI:** 10.1093/pnasnexus/pgae385

**Published:** 2024-09-06

**Authors:** Baohua Cai, Yixiang Wang, Xin Yang, Yanchen Li, Jinghao Zhai, Yaling Zeng, Jianhuai Ye, Lei Zhu, Tzung-May Fu, Qi Zhang

**Affiliations:** Shenzhen Key Laboratory of Precision Measurement and Early Warning Technology for Urban Environmental Health Risks, School of Environmental Science and Engineering, Southern University of Science and Technology, Shenzhen, Guangdong 518055, China; Shenzhen Key Laboratory of Precision Measurement and Early Warning Technology for Urban Environmental Health Risks, School of Environmental Science and Engineering, Southern University of Science and Technology, Shenzhen, Guangdong 518055, China; Shenzhen Key Laboratory of Precision Measurement and Early Warning Technology for Urban Environmental Health Risks, School of Environmental Science and Engineering, Southern University of Science and Technology, Shenzhen, Guangdong 518055, China; Provincial Observation and Research Station for Coastal Atmosphere and Climate of the Greater Bay Area, Shenzhen 518055, China; Shenzhen Key Laboratory of Precision Measurement and Early Warning Technology for Urban Environmental Health Risks, School of Environmental Science and Engineering, Southern University of Science and Technology, Shenzhen, Guangdong 518055, China; Shenzhen Key Laboratory of Precision Measurement and Early Warning Technology for Urban Environmental Health Risks, School of Environmental Science and Engineering, Southern University of Science and Technology, Shenzhen, Guangdong 518055, China; Provincial Observation and Research Station for Coastal Atmosphere and Climate of the Greater Bay Area, Shenzhen 518055, China; Shenzhen Key Laboratory of Precision Measurement and Early Warning Technology for Urban Environmental Health Risks, School of Environmental Science and Engineering, Southern University of Science and Technology, Shenzhen, Guangdong 518055, China; Provincial Observation and Research Station for Coastal Atmosphere and Climate of the Greater Bay Area, Shenzhen 518055, China; Shenzhen Key Laboratory of Precision Measurement and Early Warning Technology for Urban Environmental Health Risks, School of Environmental Science and Engineering, Southern University of Science and Technology, Shenzhen, Guangdong 518055, China; Provincial Observation and Research Station for Coastal Atmosphere and Climate of the Greater Bay Area, Shenzhen 518055, China; Shenzhen Key Laboratory of Precision Measurement and Early Warning Technology for Urban Environmental Health Risks, School of Environmental Science and Engineering, Southern University of Science and Technology, Shenzhen, Guangdong 518055, China; Provincial Observation and Research Station for Coastal Atmosphere and Climate of the Greater Bay Area, Shenzhen 518055, China; Shenzhen Key Laboratory of Precision Measurement and Early Warning Technology for Urban Environmental Health Risks, School of Environmental Science and Engineering, Southern University of Science and Technology, Shenzhen, Guangdong 518055, China; Provincial Observation and Research Station for Coastal Atmosphere and Climate of the Greater Bay Area, Shenzhen 518055, China; Department of Environmental Toxicology, University of California, Davis, CA 95616, USA

**Keywords:** brown carbon, phenols, nitrosonium ions, fast aqueous-phase dark reaction

## Abstract

Dark aqueous-phase reactions involving the nitrosation and nitration of aromatic organic compounds play a significant role in the production of light-absorbing organic carbon in the atmosphere. This process constitutes a crucial aspect of tropospheric chemistry and has attracted growing research interest, particularly in understanding the mechanisms governing nighttime reactions between phenols and nitrogen oxides. In this study, we present new findings concerning the rapid dark reactions between phenols containing electron-donating groups and inorganic nitrite in acidic aqueous solutions with pH levels <3.5. This reaction generates a substantial amount of nitroso- and nitro-substituted phenolic compounds, known for their light-absorbing properties and toxicity. In experiments utilizing various substituted phenols, we demonstrate that their reaction rates with nitrite depend on the electron cloud density of the benzene ring, indicative of an electrophilic substitution reaction mechanism. Control experiments and theoretical calculations indicate that the nitrosonium ion (NO^+^) is the reactive nitrogen species responsible for undergoing electrophilic reactions with phenolate anions, leading to the formation of nitroso-substituted phenolic compounds. These compounds then undergo partial oxidation to form nitro-substituted phenols through reactions with nitrous acid (HONO) or other oxidants like oxygen. Our findings unveil a novel mechanism for swift atmospheric nitrosation and nitration reactions that occur within acidic cloud droplets or aerosol water, providing valuable insights into the rapid nocturnal formation of nitrogen-containing organic compounds with significant implications for climate dynamics and human health.

**Significance Statement**

Phenols undergo rapid dark reactions with nitrite and HONO in acidic aqueous solutions, yielding products with notable light-absorbing properties and toxicity. This process is a critical component of nighttime chemistry in the troposphere, yet its underlying mechanism remains unclear. Our study reveals that nitrite can produce a substantial quantity of nitrosonium ions (NO^+^) within acidic cloud droplets and aerosols. These ions then react with electron-rich organic compounds in the atmosphere, leading to the formation of various nitroso and nitro compounds. This finding highlights the necessity of reassessing atmospheric aqueous-phase reaction mechanisms involved in the generation of nitrogen-containing organic compounds, which holds significant implications for both climate change and public health.

## Introduction

Organic compounds that absorb light, also known as brown carbon (BrC), constitute a crucial component of atmospheric organic aerosols (1). These compounds impact radiative forcing by absorbing sunlight, especially at shorter wavelengths, and by modifying the optical properties of black carbon (2, 3). Moreover, the presence of light-absorbing organic compounds, particularly aromatic compounds, has garnered increasing attention due to their significant environmental and health implications. These compounds are known to contribute to elevated air pollution risks and associated mortality and morbidity (4–6). Global estimates attributed ∼7 million deaths to air pollution in 2019 (7–9), underscoring the importance of studying atmospheric chemical processes that contribute to the formation of light-absorbing and toxic organic carbon (10–13).

Atmospheric aqueous-phase reactions play a pivotal role in the generation of secondary species (14–16), with a key outcome being the formation of light-absorbing organic carbon (1, 17). Nitrogen-containing aromatic compounds, particularly phenols, are the main contributors to chromophores in BrC (18). While numerous studies have investigated aqueous-phase reactions for the production of nitrogen-containing phenols under light irradiation in laboratory settings (19–27), a notable gap remains in our understanding due to limited research conducted under dark conditions typical of nighttime environments.

Kroflič et al. (28) showed that guaiacol (GUA) reacts with sodium nitrite under dark conditions in a solution with a pH of 4.5. However, this reaction proceeded slowly, with ∼20% of GUA remaining unreacted after 24 h. In a follow-up study, quantum chemistry was employed to demonstrate that the potential mechanism of this reaction involves the formation of ·NO and ·NO_2_ radicals via the thermal decomposition of HONO. These radicals then react with GUA to yield 4-nitroguaiacol and 6-nitroguaiacol (29). Furthermore, a similar experimental study utilized electrochemical techniques to investigate the potential mechanism of the dark aqueous-phase nitration of 3-methylcatechol by NO_2_^−^ at a higher pH of 6.5 (30). Their results indicate that NO_2_^−^ undergoes an addition reaction with 3-methyl-o-quinone, which is a reactive intermediate, leading to the formation of methyl-nitrocatechol compounds (31).

It is worth noting that most documented dark aqueous-phase reactions were conducted at pH >4.5, while the pH of actual atmospheric aqueous phases can be considerably lower. For example, the pH of clouds and fog water ranges from 2.0 to 7.0, while continental aerosols display a pH range of −1.0 to 5.0. In urban areas, where concentrations of aromatic compounds are elevated due to anthropogenic emissions or biomass burning, aerosols typically maintain a pH <4.0 (32–39). Recently, Wang et al. (40) reported a fast dark aqueous-phase reaction between catechol and nitrite at pH 3.5 that achieved nearly complete catechol conversion within 2 h. However, the specific mechanism underlying the rapid reaction between phenols and nitrite at low pH levels remains unknown.

To address this knowledge gap, we investigated dark aqueous-phase reactions between nitrite and different phenols under acidic atmospheric conditions. Additionally, we conducted quantum chemistry calculations to gain insights into the reactions between phenols and nitrosonium ions (NO^+^). Our results reveal that in low-pH aqueous solutions, nitrite mainly exists as the nitrous acidium ion (H_2_ONO^+^), which converts into NO^+^. This reactive species promptly reacts with phenolates in the absence of light, leading to a substantial production of light-absorbing organic carbon.

## Results and discussion

### Fast aqueous-phase dark reaction of phenols with inorganic N(III) Species

We studied the reactions involving seven different phenols and NaNO_2_ using the experimental conditions outlined in Table S1, SI Appendix. In aqueous solutions containing nitrite, three inorganic N(III) species may coexist: the nitrous acidium ion (H_2_ONO^+^), nitrous acid (HONO), and nitrite (NO_2_^−^) (41–44). The mole fractions of these species are influenced by the pH value of the solution (41).

**Fig. 1.**
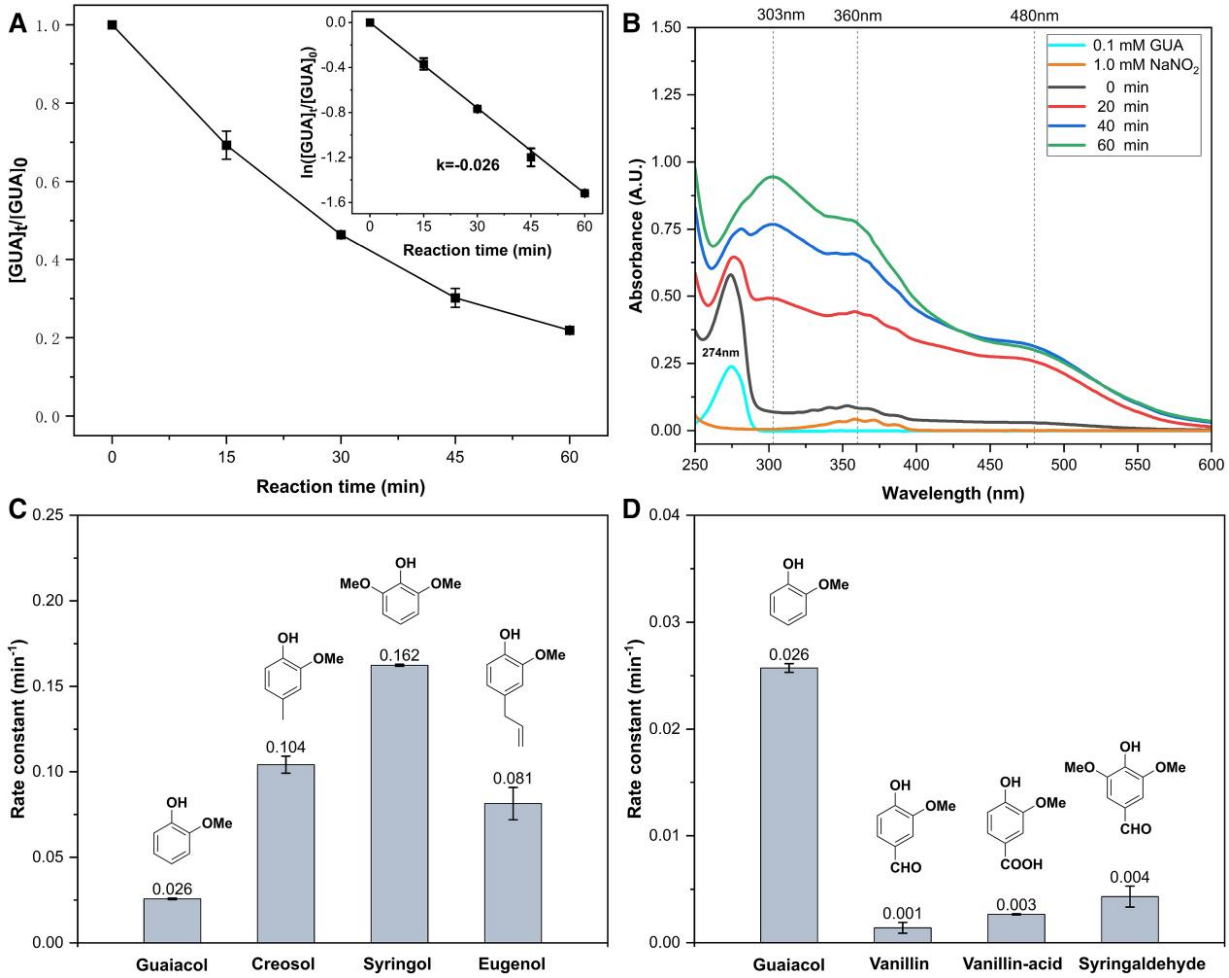
A) The kinetics of the dark aqueous-phase reaction between GUA and NaNO_2_, with the inset showing the pseudo-first-order rate constant for the reaction. B) UV–Vis absorption spectra of the reaction solution containing GUA and NaNO_2_ at different time points during the reaction. C) and D) Pseudo-first-order rate constants for various substituted GUAs undergoing dark aqueous reactions with NaNO_2_. Experimental conditions: [phenols] = 0.1 mM, [NaNO_2_] = 1 mM, pH = 3.0 ± 0.1, with zero air bubbling, room temperature.

Figure 1A shows the aqueous reaction kinetics of 0.1 mM GUA and 1 mM NaNO_2_ in a pH = 3.0 (±0.1) solution under dark conditions with air bubbling. GUA is a common volatile methoxyphenol in the atmosphere, primarily derived from biomass burning (45, 46). In this experimental setup, the reaction between GUA and NaNO_2_ exhibited first-order kinetics, with a pseudo-first-order rate constant of ∼0.026 min^−1^ and a half-life time of ∼27 min. As a result, ∼89% of the initial GUA was consumed within 1 h. The reaction generated a significant amount of light-absorbing organic carbon, including 4-NGUA (4-nitrosoguaiacol) and 6-NGUA (6-nitrosoguaiacol). This is demonstrated by notable absorption peaks observed at 303, 360, and 480 nm (Fig. 1B). The formation kinetics of 4-NGUA and 6-NGUA, along with the degradation rate of GUA, aligns with the formation rate of the nitro-substituted GUA products (Figs. S2 and S3, SI Appendix).

The kinetics of dark aqueous-phase reactions between GUA and inorganic N(III) species was further examined under varying molar ratios of GUA and NaNO_2_ that are relevant to atmospheric conditions. Concentrations of methoxyphenols in biomass burning plumes can reach several micrograms per cubic meter (47). Due to their substantial Henry's law constants (48), methoxyphenols readily dissolve in atmospheric aqueous phases, with a total concentration reaching as high as 0.1 mM in fog waters (49–51). Nitrite concentrations in cloud and fog droplets range from 0.01 to 1000 μM (52, 53). As shown in Fig. S4, SI Appendix, when the NaNO_2_ concentration greatly exceeds that of GUA (at a molar ratio >10:1), the reaction follows pseudo-first-order kinetics, with the rate constant directly proportional to the nitrite concentration. Under these conditions, the GUA decay rate (*R* = *k*_obs_ × [GUA]_0_) remains constant at ∼0.0026 mM min^−1^ with an initial NaNO_2_ concentration of 1.0 mM (Fig. S5, SI Appendix). However, when the initial concentrations of GUA and nitrite are comparable, the reaction exhibits second-order kinetics (Fig. S6, SI Appendix).

We further investigated the reaction rates between different substituted phenols and nitrite, using additional phenols commonly found in the atmosphere (45, 46). These phenols encompass those substituted with electron-donating groups, such as creosol, syringol, and eugenol, as well as those with electron-withdrawing groups, such as vanillin, vanillin acid, and syringaldehyde (Fig. S1 and Table S1, SI Appendix). The pseudo-first-order rate constants for the decay of these phenols exhibited a notable increase in the presence of electron-donating groups (Fig. 1C) and conversely decreased with the inclusion of electron-withdrawing groups (Fig. 1D). This observed trend underscores a strong correlation between substituent properties and system reactivity, indicating that the electron cloud density of the benzene ring plays a crucial role in determining the pseudo-first-order rate constant in the dark aqueous-phase reaction between phenols and nitrite under low pH conditions. Specifically, a higher electron cloud density accelerates the reaction rate, while a lower electron cloud density retards it. These findings support an electrophilic substitution mechanism, where an aromatic ring with higher electron density is more susceptible to electrophilic attack by the inorganic N(III) species, resulting in a faster reaction rate.

### Potential mechanisms for aqueous reactions of phenols and nitrite at low pH

**Fig. 2.**
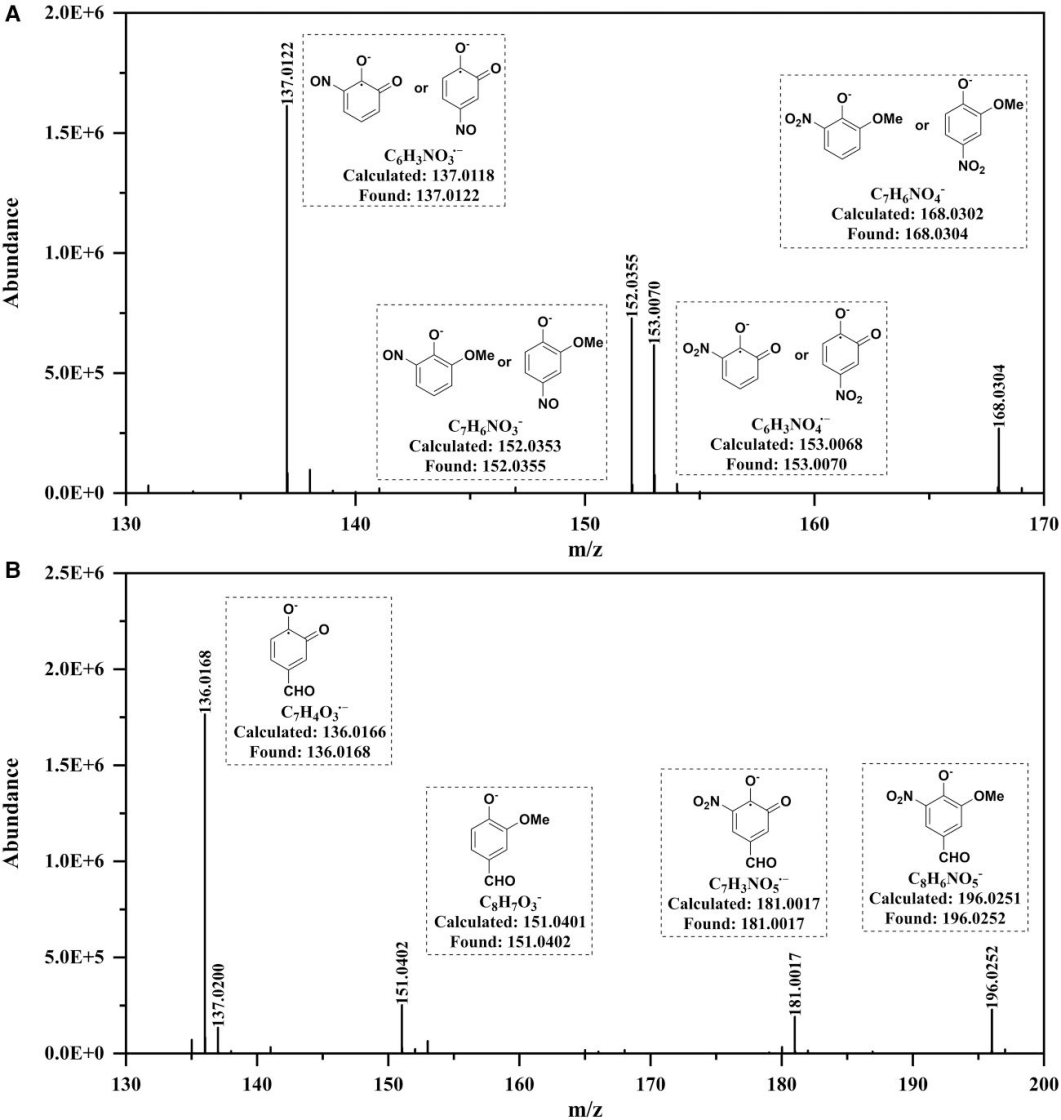
The HRMS of the reaction products of phenolic compounds and NaNO_2_. A) GUA. B) Vanillin. Experimental conditions: [phenols] = 0.1 mM, [NaNO_2_] = 1 mM, pH = 3.0 ± 0.1, zero air bubbling, room temperature.

To elucidate the mechanisms of dark aqueous-phase reaction between phenols with nitrite, we used high-resolution mass spectrometry (HRMS) to analyze the molecular compositions of the reaction products. Figure 2A shows the negative-mode electrospray ionization (ESI) mass spectra (MS) of the solution containing 0.1 mM GUA and 1 mM NaNO_2_ at pH 3.0 after 30 min in the dark. Alongside the ion corresponding to nitro-GUA at *m/z* = 168.0304 (C_7_H_6_NO_4_^−^), we also detected an ion representing nitroso-GUA at *m/z* = 152.0355 (C_7_H_6_NO_3_^−^), which indicate the occurrence of nitroso substitution reaction. In contrast, the reaction between nitrite and vanillin proceeded slowly (Fig. 2B), and analysis of its HRMS data did not reveal ions indicative of nitroso-substituted vanillin. Instead, only nitro-substituted vanillin ions (e.g. C_8_H_6_NO_5_^−^ at *m/z* = 196.0252) were identified. These findings suggest that the rapid reactions observed for electron-rich phenols with nitrite in acidic aqueous environments were primarily facilitated by the nitrosation pathway.

The reaction mechanism between phenols and nitrite in the aqueous phase is known to occur under two distinct conditions. One of them involves an addition reaction that takes place at pH above 5.5, where NO_2_^−^ is the predominant reactive nitrogen species (RNS) driving the reaction (30, 31). As illustrated in Fig. S7A, SI Appendix, for catechol, upon oxidation by HONO, the catechol molecule releases two hydrogens and two electrons, forming diquinone as an intermediate product. Subsequently, diquinone undergoes an addition reaction with NO_2_^−^, leading to the formation of nitro-substituted products. The other mechanism involves radical reactions occurring at pH levels <5.5, where the ·NO radical and ·NO_2_ radical, generated through the thermal decomposition or oxidation of HONO, act as the main RNS that interact with phenols (28, 29, 54–57). Within this solution, phenols (Ar–OH) undergo hydrogen-atom abstraction by free radicals (e.g. ·OH, ·NO, and ·NO_2_) to form corresponding phenoxy radicals (Ar–O), which subsequently isomerize into aryl radicals (·Ar–OH). These ·Ar–OH radicals then react with ·NO and ·NO_2_ to yield nitro-substituted and nitroso-substituted products, respectively (Fig. S7B, SI Appendix).

In this study, we discovered that although the reactions between phenols and nitrite in solutions with pH levels >3.5 align with these mechanisms, they do not fully explain the experimental observations at pH < 3.5. This is particularly evident in comparing the reaction rates of different phenols. For example, the pseudo-first-order reaction rate between GUA and nitrite was ∼26 times faster than that between vanillin and nitrite (Fig. 1D), indicating a pronounced dependence of the reaction rate on the electron cloud density of the benzene ring. However, considering our earlier observation of a rapid reaction between vanillin and the ·NO_2_ radical at pH 3.0 (20), the presence of electron-withdrawing functional groups on the benzene ring did not significantly reduce the nitration rate of phenols. Thus, it appears that that the nitrosation process, rather than nitration, is considerably affected by the electron density of the benzene ring. This conclusion is further supported by the absence of nitroso-vanillin in the reaction products of vanillin and nitrite as determined through HRMS analysis.

Here, we propose a novel mechanism suggesting that the reaction between phenols and nitrite at pH < 3.5 proceeds through the interaction of phenols with NO^+^. In aqueous solutions, nitrite can exist in three forms—H_2_ONO^+^, HONO, and NO_2_^−^ (41–44). The p*K*a values for H_2_ONO^+^ and HONO are 1.7 and 2.8, respectively (41). As illustrated in the distribution curves of these N(III) species relative to pH (Fig. S8, SI Appendix), H_2_ONO^+^ dominates with mole fractions exceeding 0.8 at pH <1.0. However, at pH > 3.5, H_2_ONO^+^ is depleted, and NO_2_^−^ and HONO become the predominant forms. As the pH drops below 3.5, the proportion of H_2_ONO^+^ increases, facilitating the release of NO^+^ by H_2_ONO^+^.

To further explore this mechanism and gain insights into the key role of NO^+^, we conducted quantum chemistry calculations to probe the reactions between NO^+^ and three phenols: GUA, vanillin (consisting of a GUA moiety with an electron-withdrawing aldehyde functional group linked to the ring), and creosol (containing a GUA moiety with an electron-donating methyl functional group attached to the ring).

In a previous report, the reaction mechanism between GUA and NO^+^ was meticulously investigated, yet no plausible transition state (TS) could be identified (29). Similarly, our investigation into the reaction mechanisms of NO^+^ with vanillin and creosol also failed to yield viable TSs. Details regarding TS calculations and the calculated energy barriers for the reactions are given in Section S1 and Figs. S9 and S10, SI Appendix. In essence, these findings indicate that there are no discernable reactions between NO^+^ and GUA, vanillin, and creosol in their molecular forms.

**Fig. 3.**
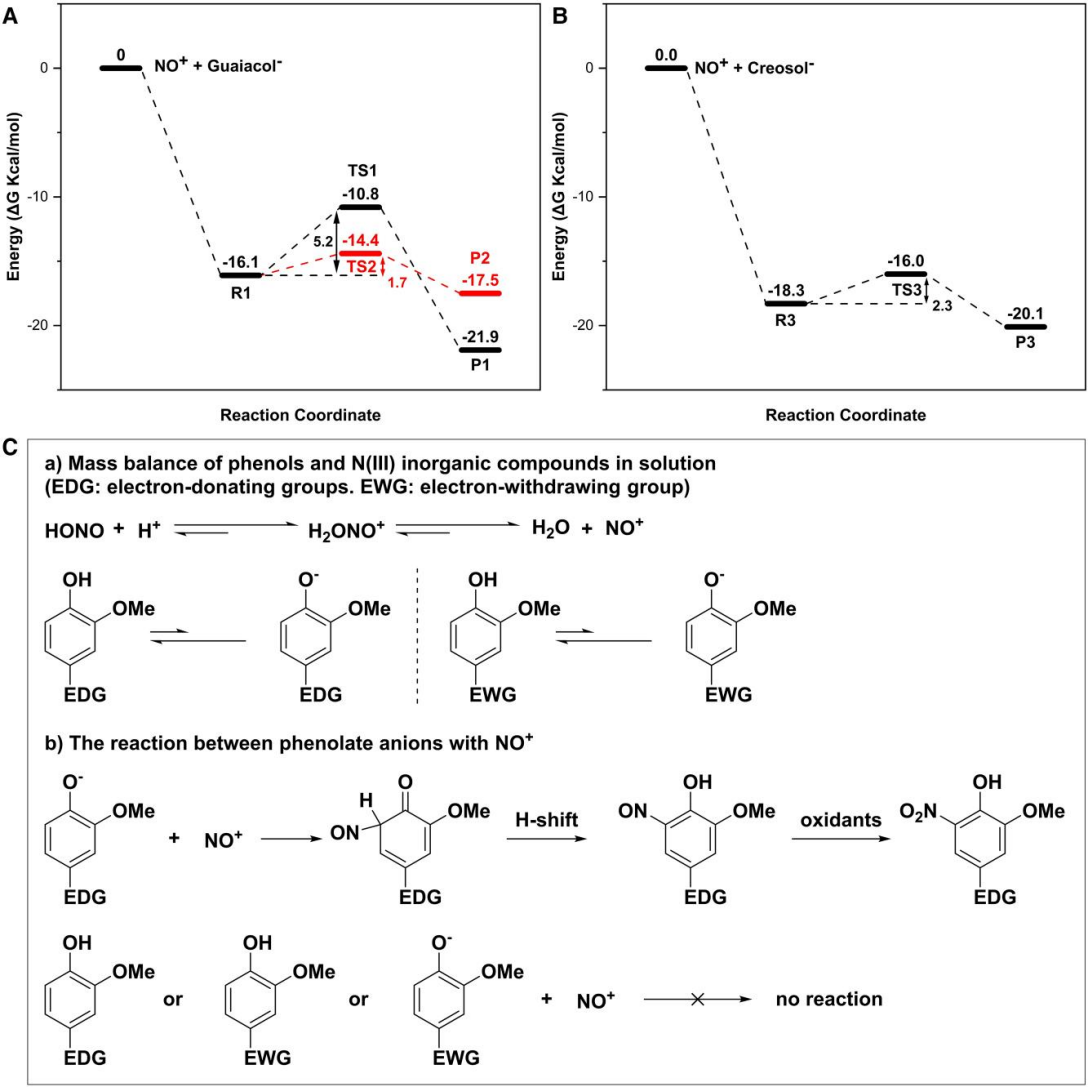
Gibbs free energy (in kcal/mol at 298.15 K) profiles for the reaction of phenolic compounds and NO^+^ at the DLPNO-CCSD(T)/aug-cc-pVTZ/SMD(water)//B3LYP-D3(BJ)/aug-cc-pVTZ/SMD(water) level of theory with the zero-point energy correction applied. A) GUA, the black line represents the reaction mechanisms for forming 4-NGUA and the red line represents the reaction mechanisms for forming 6-NGUA, B) Creosol. C) The new reaction mechanism of phenols and N(III) inorganic compounds. The molecular configurations along the reaction coordinates in A) and B) are presented in Fig. S9.

However, as illustrated in Fig. 3A and B, the activation energies for the reactions of the anions of GUA (C_7_H_7_O_2_^−^) and creosol (C_8_H_9_O_2_^−^) with NO^+^ are notably low (5.2, 1.7, and 2.3 kcal/mol, respectively), indicating the potential for rapid electrophilic substitution reactions between NO^+^ and GUA and creosol. The molecular structures of the reaction intermediates and products are presented in Fig. S11, SI Appendix. In contrast, no viable TS has been identified for the vanillin system (C_8_H_7_O_3_^−^) (Section S1 and Fig. S10D, SI Appendix), which is consistent with the slow reaction observed in the experiment involving vanillin and NaNO_2_ at pH 3.0 (Fig. 1D). These results, coupled with the lack of nitroso-vanillin as a reaction product, indicate that phenols with electron-withdrawing groups are not prone to rapid reactions with NO^+^.

Figure 3C summarizes the novel reaction mechanism we propose for dark aqueous reactions involving phenols and N(III) species at pH < 3.5. The initial step of this mechanism involves the release of NO^+^ from H_2_ONO^+^, which is the primary form of the N(III) species under low pH conditions. It subsequently undergoes an electrophilic substitution reaction with the phenolate anions (Ar–O^−^), leading to the formation of nitroso-substituted products. These products are then partially oxidized to nitro-substituted products by HONO or other oxidants, such as oxygen (Fig. 3C). Our experimental findings and theoretical calculations indicate that this reaction pathway is slow for phenols containing electron-withdrawing groups, even under low pH conditions. This observation aligns with previously reported mechanisms (28–31).

**Fig. 4.**
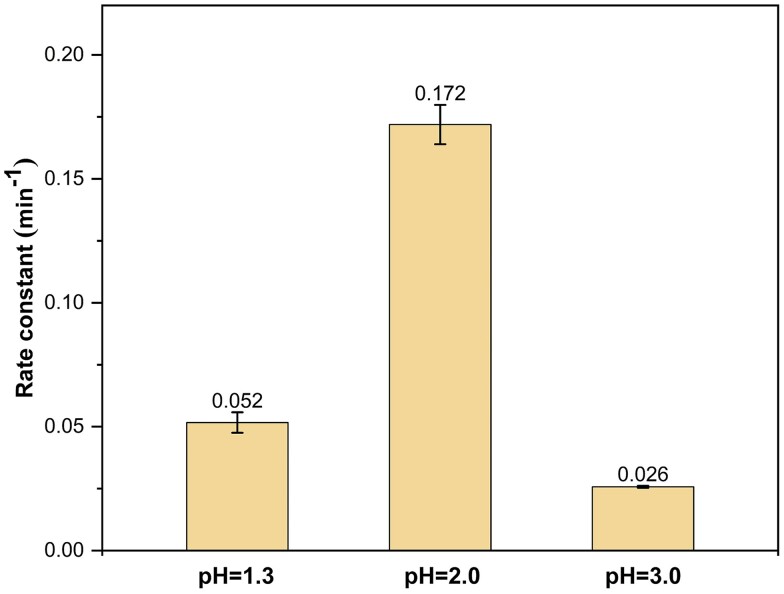
Effect of acidic conditions (pH < 3.0) on the pseudo-first-order rate constants of dark aqueous-phase reactions involving GUA and NaNO_2_. Experimental conditions: [GUA] = 0.1 mM, [NaNO_2_] = 1 mM, and zero air bubbling.

In this newly proposed mechanism, the reaction rate is influenced by two factors: (ⅰ) the concentrations of NO^+^ and the phenolate anion and (ⅱ) the activation energy associated with the reaction between NO^+^ and the phenolate anion. In theory, as the pH value decreases <3.0, the concentration of NO^+^ is expected to rise (Fig. S8, SI Appendix), while the concentration of the phenolate anion is anticipated to decrease. This intricate balance could lead to a rate constant that initially rises and then declines with decreasing pH values. This theoretical prediction is supported by the observed variations in the pseudo-first-order rate constants of GUA and NaNO_2_ at pH values of 1.3, 2.0, and 3.0, where *k*_pH = 2.0_ > *k*_pH = 1.3_ > *k*_pH = 3.0_ (Fig. 4).

We further demonstrate the applicability of this mechanism in predicting dark aqueous-phase reactions involving other phenols and nitrite, as it not only explains the rapid dark aqueous-phase reactions of methoxyphenol and nitrite but also sheds light on the fast reaction between diphenol (catechol) and nitrite (40). As illustrated in Fig. S10, SI Appendix, after a proton loss from catechol, the resulting catechol anion can react with NO^+^ with low activation energies of 4.3 and 1.3 Kcal/mol (Fig. S13, SI Appendix, structures of the intermediates and products are presented in Fig. S14, SI Appendix). Furthermore, Figs. S5–S17, SI Appendix, depict the TSs and activation energies for the reaction between 4-chloro-2-methoxyphenol (4ClGUA) and NO^+^, predicting that 4ClGUA can rapidly react with nitrite in the dark aqueous phase despite the weak electron-withdrawing nature of the chlorine atom. Our subsequent experiments confirmed this prediction, showing a pseudo-first-order rate constant of ∼0.028 min^−1^ and a half-life time of ∼25 min for the reaction between 4ClGUA and nitrite (Fig. S18, SI Appendix).

### Effects of atmospheric conditions

**Fig. 5.**
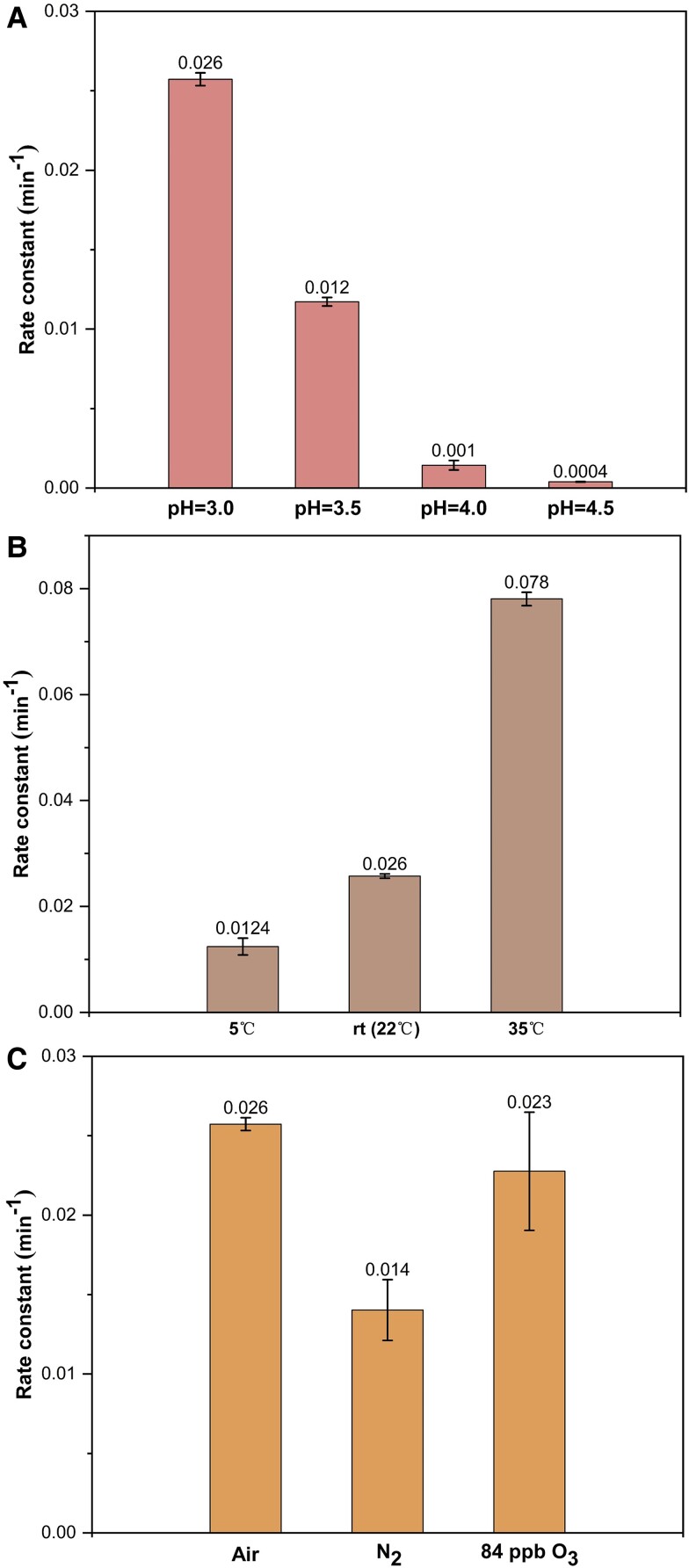
Dark aqueous-phase reaction rates of GUA and NaNO_2_ under various atmospheric conditions: A) Different pH values. B) Different temperatures. C) Different air bubbling. Experimental conditions: [GUA] = 0.1 mM, [NaNO_2_] = 1 mM, pH = 3.0 ± 0.1 (unless explicitly stated), with zero air bubbling (unless explicitly stated).

To assess the reaction rates of phenols with nitrite under different atmospheric conditions, we conducted a series of control experiments using GUA and NaNO_2_ as a model reaction system (Table S1, SI Appendix). We studied the influence of solution pH, recognizing its pivotal role in tropospheric aqueous-phase chemistry, which includes its influences on processes such as gas–liquid-phase partitioning and ion speciation of dissolved compounds (35, 36). The dark reaction between GUA and NaNO_2_ exhibited rapid kinetics at pH values <3.5, with half-lives of <58 min, while the reactivity greatly decreased above a pH of 4.0 (Fig. 5A). Additionally, a temperature-dependent relationship was evident, with higher temperatures markedly accelerating the reaction rate (Fig. 5B). These findings indicate that rapid dark aqueous-phase reactions of phenols and nitrite are more likely to occur under conditions of elevated temperature and low pH.

Figure 5C shows that the dark aqueous reactions of GUA and NaNO_2_ are facilitated by the presence of dissolved oxygen. In our experiments, we simulated an anoxic environment similar to the upper atmosphere by bubbling inert gas (N_2_) through the solution. Under anoxic conditions, we observed a pseudo-first-order rate constant of ∼0.014 min^−1^ and a half-life of ∼50 min at pH = 3.0, which are nearly 50% slower than the rates observed under air-saturated conditions. However, elevated ozone concentration was found to have minimal effects on the reaction rate. Specifically, we observed a rate constant of ∼0.023 min^−1^ and a half-life of ∼30 min at an ozone concentration of 84 ppb, which were similar to those in air-saturated solutions. This suggests that ozone concentration within typical ambient levels does not significantly impact the reaction rate.

### Implications

Inorganic N(III) species are frequently detected in atmospheric cloud droplets and aerosols, existing in diverse forms across a wide range of pH values. This diversity influences their chemical properties and underscores their significance as sources of RNS. Chemical interactions between VOCs and RNS components can potentially lead to the formation of nitroso and nitro compounds, which have strong light-absorbing properties and contribute to the complex chemistry and radiative forcing within the atmosphere. Additionally, nitro and nitroso compounds are associated with considerable toxicity.

**Fig. 6.**
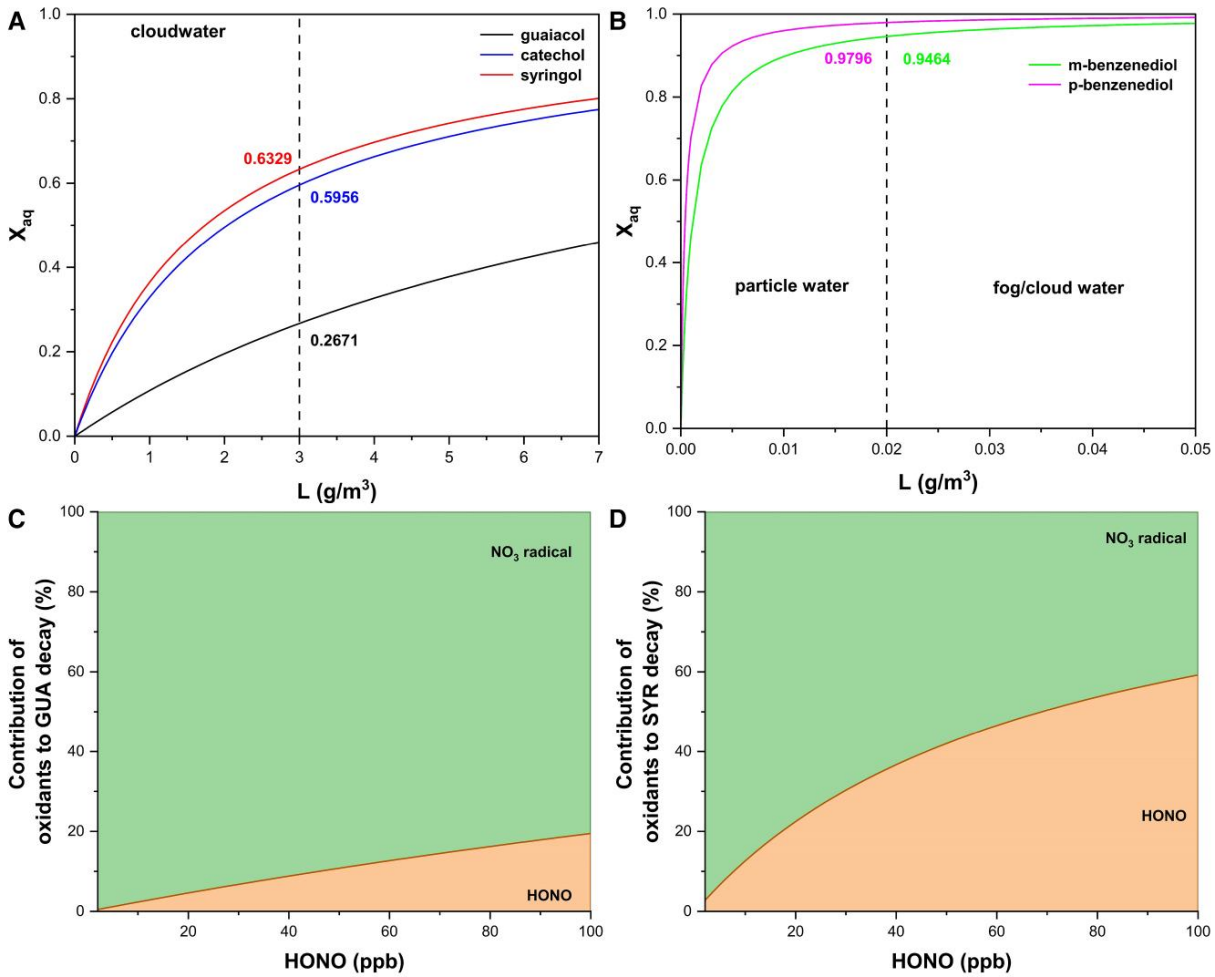
The gas–water distribution of different phenolic compounds under an air temperature of 5 °C and varying LWCs: A) compounds with lower Henry's law constants (<10^6^ m atm^−1^) and B) compounds with Henry's law constants (>10^6^ m atm^−1^). The percent contributions of NO_3_ radicals and HONO to the degradation of GUA (C) and syringol (D) as a function of HONO gas-phase concentrations ([HONO]_g_) at an aqueous-phase pH = 3.0.

In cloud droplets and aerosol particles, light-absorbing material primarily originates from secondary formation processes and biomass burning, which collectively contribute to 67–85% of the total BrC content (17). Phenolic compounds are significant products of biomass burning, with global emissions estimated at 4.7 Tg per year (45, 46). Due to their varying substitution structures, the Henry's law constants for these compounds range from 4 × 10¹ to 3.1 × 10^8^ M atm^−1^ (48). We selected GUA, catechol, syringol, m-benzenediol, and p-benzenediol to model gas–water distribution (Section S3, SI Appendix). Our simulations indicate that for phenols with lower Henry's law constants (<10^6^ M atm^−1^), up to 60% of syringol and catechol, and about 26% for GUA, may partition into the cloud water phase under the liquid water content (LWC) condition of 3 g m^−3^ (Fig. 6A). Conversely, for phenols with a high Henry's law constant (>10^6^ M atm^−1^), nearly all of the compounds partition into the aqueous phase under fog/cloud conditions (e.g. LWC > 0.02 g m^−3^), and a significant proportion of these compounds distribute into the aerosol water phase even when LWC is as low as a few μg m^−3^ (Fig. 6B).

During the daytime, photochemical oxidation of phenolic compounds in the aqueous phase, primarily driven by OH radicals, singlet oxygen (^1^O_2_*), and triplet carbonyls (³C*) (58, 59), is an important source of secondary organic aerosol (60). In the presence of inorganic N(III) species, additional RNS like ·NO and ·NO_2_ are generated, which can react with phenolic compounds to form light-absorbing substances (20, 25, 27).

The significant role of aqueous-phase secondary processes in accelerating the formation of light-absorbing substances has been observed during nighttime as well (61). However, previous experimental studies have indicated that the dark aqueous-phase formation of nitrophenols occurs slowly (28, 30). In this study, we have identified a fast reaction mechanism between nitrite and phenols within the dark aqueous solution under conditions relevant to the atmosphere. This discovery may shed light on the rapid formation of BrC observed during nighttime. It represents a noteworthy reaction mechanism, particularly relevant in regions where the pH of atmospheric water droplets and aerosols has shown a trend of persistent decline (37, 39).

At nighttime, the degradation of phenols is primarily driven by NO_3_ radicals and HONO (62, 63). Field observations of nighttime biomass burning plumes reveal high HONO concentrations, potentially reaching up to 100 ppb (40). In contrast, NO_3_ concentrations are relatively low (<3 ppt), with NO_3_ primarily reacting with NO_2_ to form N_2_O_5_ and engaging in gas-phase reactions with biomass burning volatile organic compounds (64). To better understand the contributions of HONO and NO_3_ radicals to the aqueous-phase degradation of phenolic compounds at nighttime, we employed a box model (Section S3, SI Appendix). The typical nighttime concentration of NO_3_ radicals in cloud droplets is around 10^−12^ M (62), while HONO concentrations in the aqueous phase depend directly on its gas-phase levels. We simulated gas-phase HONO concentrations ranging from 0 to 100 pb. Our results show that at 100 ppb [HONO]_g_, ∼20% of GUA is degraded by aqueous-phase HONO at pH 3.0 (Fig. 6C), while for syringol, this figure rises to 59% (Fig. 6D). For other electron-rich phenolic compounds, the proportion of aqueous-phase degradation driven by HONO could be even higher. These findings underscore the critical role of HONO in the nighttime degradation of phenolic compounds and highlight the importance of considering this pathway in atmospheric chemistry models.

It is also important to note that the core RNS (NO^+^) identified in this reaction mechanism could participate in reactions with other nucleophilic compounds present in the atmosphere, such as amines. These reactions might lead to the generation of nitrosamines and related toxic compounds, posing significant implications for human health. Future studies on atmospheric aqueous-phase reactions may need to focus on enhancing our understanding of the chemical behavior of inorganic N(III) species under acidic conditions. This knowledge is crucial for unraveling the complexity of atmospheric chemistry and its wide-ranging impacts on the environment and human health. Specifically, further investigation into the mechanisms and kinetics of dark aqueous-phase reactions involving inorganic N(III) species under low pH conditions will provide valuable insights into the formation of secondary N-containing organic compounds and their potential impacts on air quality, climate, and public health. Moreover, such research can aid in the development of targeted strategies aimed at mitigating the impacts of these reactions on atmospheric composition and chemistry.

## Materials and methods

### Materials

Organic reagents used in this study are outlined in Fig. S1, SI Appendix. GUA (99%), 4ClGUA (98%), Na_2_SO_4_ (>99%), and NaNO_2_ (>99%) were purchased from Macklin. Vanillin acid (98%), eugenol (98%), syringaldehyde (98%), creosol (98%), and syringol (98%) were purchased from Meryer. Vanillin (98%) was purchased from Aladdin.

### Characterization methods

#### HPLC analysis

The concentration of the precursors was determined using an HPLC (Thermo Scientific UltiMate 3000) equipped with a diode array detector and an Agilent 5 TC-C18 column (150 × 4.60 mm, 5 μm). The column temperature was maintained at 25 °C, and the flow rate was set to 1 mL/min. Detection was performed at a wavelength of 274 nm. To analyze GUA, eugenol, syringol, creosol, and 4ClGUA , the mobile phase consisted of 60/40 (v/v) acetonitrile/water acidified with trifluoroacetic acid (TFA, 0.1%). To analyze vanillin, vanillin acid, and syringaldehyde, the mobile phase consisted of 50/50 (v/v) methanol/water acidified with TFA (0.1%).

#### Direct infusion HRMS

The reaction solution was filtered through the filter membrane and then directly introduced into the Agilent 6546 quadrupole time-of-flight mass spectrometer (QTOF-MS, Santa Clara, CA, USA) with ESI source in negative mode. The MS parameters were applied as follows: nebulizer, 25 psi; gas flow, 10 L/min; sheath gas temperature, 330 °C; capillary voltage, 3,500 V; and sheath gas flow, 12 L/min. The MS fragmentation data were collected in the 90–500 m/z range. MassHunter Qualitative and Quantitative software version 10.0 was used for data analysis.

#### UV–Vis spectroscopy

An ultraviolet–visible spectrophotometer (Youke, T2602, Shanghai, China) was used to monitor the absorption of GUA during its reactions with NaNO_2_ and to determine the spectrum of samples in 300–600 nm. The reaction solution was directly loaded into the instrument before irradiation without any alterations. Spectra of the GUA—NaNO_2_ reaction were collected every 20 min for 1 h.

#### Dark bulk aqueous experiment

All experiments were performed in a 250-mL airtight Pyrex tube which was wrapped in aluminum foil equipped with a magnetic stirrer and a bubble tube for feeding high-purity zero air or nitrogen. A 200-mL reaction solution of the organic compound and NaNO_2_ with other reactants was prepared. The pH of the reaction solution was regulated by H_2_SO_4_ and NaOH. The pH of solutions was measured with a pH meter/redox potentiometer/conductivity meter (AZ-86555) that was calibrated with commercial pH standards. Aliquots of 10 mL reaction solution, to which 200 μL of 1 M NaOH aqueous solution was added to stop the reaction, were sampled for chemical analysis every 15 min for 1 h. Each experiment was repeated at least twice.

#### Quantum chemistry calculations

Geometry optimizations for all molecular structures of the reactants, products, and TSs were using the B3LYP-D3(BJ) (65, 66) (B3LYP with the GD3BJ dispersion correction) functional with the aug-cc-pVTZ basis set (67) using the SMD solution model to calculate solvent effects in water (68) with Gaussian 16 package (69). Optimized structures were verified by frequency computations as minima (zero imaginary frequencies) or transition structures (a single imaginary frequency). Intrinsic reaction coordinate calculations were performed to ensure that the first-order saddle points found were true TSs connecting the reactants and the products. Single-point energies and solvent effects in water were calculated at DLPNO-CCSD(T)/aug-cc-pVTZ (70–75) level of theory based on the optimized geometries at the B3LYP-D3(BJ)/aug-cc-pVTZ using the SMD solvation model with the ZPE correction employed which using the ORCA 5.0.3 program package (76). The Multiwfn 3.8 (77) and Shermo 2.4 (78) were used for data analysis.

## Supplementary Material

pgae385_Supplementary_Data

## Data Availability

The data that support the findings of this study are available in the Supplementary material of this article.
