# Supplementary material for: Rapid aqueous-phase dark reaction of phenols with nitrosonium ions: Novel mechanism for atmospheric nitrosation and nitration at low pH
Source: PNAS Nexus. 2024 Sep 6;3(9):pgae385. doi: 10.1093/pnasnexus/pgae385 (PMC11410049; doi:10.1093/pnasnexus/pgae385)
Supplement: pgae385_Supplementary_Data [file pgae385_supplementary_data.pdf]

Supporting Information for

Rapid aqueous-phase dark reaction of phenols with nitrosonium ions: novel mechanism for atmospheric nitrosation and nitration at low pH

Baohua Cai[a], Yixiang Wang[a], Xin Yang[a,b], Yanchen Li[a], Jinghao Zhai[a,b], Yaling Zeng[a,b], Jianhuai Ye[a,b], Lei Zhu[a,b], Tzung-May Fu[a,b], and Qi Zhang[c]

Author affiliations: [a]Shenzhen Key Laboratory of Precision Measurement and Early Warning Technology for Urban Environmental Health Risks, School of Environmental Science and Engineering, Southern University of Science and Technology, Shenzhen 518055, China, Guangdong; [b]Provincial Observation and Research Station for Coastal Atmosphere and Climate of the Greater Bay Area, Shenzhen 518055, China; and [c]Department of Environmental Toxicology, University of California, Davis, California 95616, United States

* Xin Yang and Qi Zhang.

Email: yangx@sustech.edu.cn and dkwzhang@ucdavis.edu

This PDF file includes:

- Supporting text
- Figures S1 to S18
- Tables S1 to S2
- SI References

Supplementary Information Text

S1. Transition State Calculation. To obtain the approximate geometry for the plausible transition state of the reaction between phenols and NO^+, a modredundant calculation was employed, incrementally shortening the distance between the two atoms involved: the N atoms of NO^+ and the C atoms of the benzene ring. The distance between the two atoms involved in the N atoms of NO^+ and the C atoms of the benzene ring was incrementally shortened through a modredundant calculation. The modredundant calculation abbreviates the distance between two specified atoms, performs a geometry optimization, and subsequently extracts the energy of that structure. The proposed geometry of transition states for this system is defined as the configuration that yields the maximum electronic energy by virtue of the distance between the two bond-forming/breaking atoms. The absence of the maximum electronic energy indicates that no reasonable transition states. The calculations were carried out using the B3LYP-D3(BJ) (1, 2) (B3LYP with the GD3BJ dispersion correction) functional with the 6-311+g(d,p) basis set using the SMD solution model to calculate solvent effects in water (3) with Gaussian 16 package (4). DFT-optimized structures are illustrated using CYLView (5).

S2. Kinetic analyses.

Pseudo-First-Order analyses. According to Atkins' Physical Chemistry, 11th Edition, the reactions were identified as being pseudo first order in GUA when the initial concentration of $NaNO_2$ is much greater than the initial concentration of GUA ($[NaNO_2]_0 \gg [GUA]_0$), could be described with a pseudo-first-order rate constant:

$$\ln\frac{[GUA]_t}{[GUA]_0} = -k^I t$$

$$t_{1/2} = \frac{\ln 2}{k^I}$$

where $[GUA]_t$ is the concentration of GUA at reaction time t. $[GUA]_0$ is the initial concentration of GUA. k^I is the pseudo-first-order rate constant. $t_{1/2}$ is the half-life of GUA.

Pseudo-Second-Order analyses. The reactions were identified as pseudo second order with respect to GUA when the initial concentrations of $NaNO_2$ and GUA were similar. ($[NaNO_2]_0 \approx [GUA]_0$), could be described with a pseudo-second-order rate constant:

When $[NaNO_2]_0 = [GUA]_0$

$$\frac{1}{[GUA]_t} - \frac{1}{[GUA]_0} = k^{II} t$$

$$t_{1/2} = \frac{1}{k^{II} \times [GUA]_0}$$

where $[GUA]_t$ is the concentration of GUA at reaction time t. $[GUA]_0$ is the initial concentration of GUA. k^{II} is the pseudo-second-order rate constant. $t_{1/2}$ is the half-life of GUA.

When $[NaNO_2]_0 \neq [GUA]_0$

$$\frac{1}{[GUA]_0 - [NaNO_2]_0}\ln\frac{[GUA]_t}{[NaNO_2]_t} = k^{II} t + C$$

where $[GUA]_t$ is the concentration of GUA at reaction time t. $[NaNO_2]_t$ is the concentration of $NaNO_2$ at reaction time t. $[GUA]_0$ is the initial concentration of GUA. $[NaNO_2]_0$ is the initial concentration of $NaNO_2$. k^{II} is the pseudo-second-order rate constant.

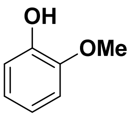

Guaiacol
Molecular Formula: $C_7H_8O_2$

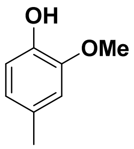

Creosol
Molecular Formula: $C_8H_{10}O_2$

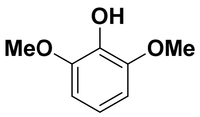

Syringol
Molecular Formula: $C_8H_{10}O_3$

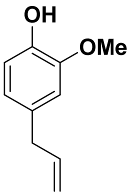

Eugenol
Molecular Formula: $C_{10}H_{12}O_2$

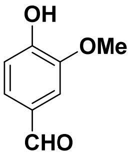

Vanillin
Molecular Formula: $C_8H_8O_3$

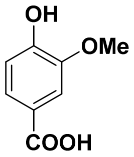

Vanillin-acid
Molecular Formula: $C_8H_8O_4$

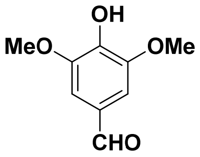

Syringaldehyde
Molecular Formula: $C_9H_{10}O_4$

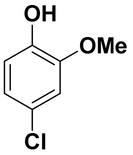

4-chloro-2-methoxyphenol
Molecular Formula: $C_7H_7ClO_2$

Figure S1. The chemical structures, name, and molecular formula for organic reagents.

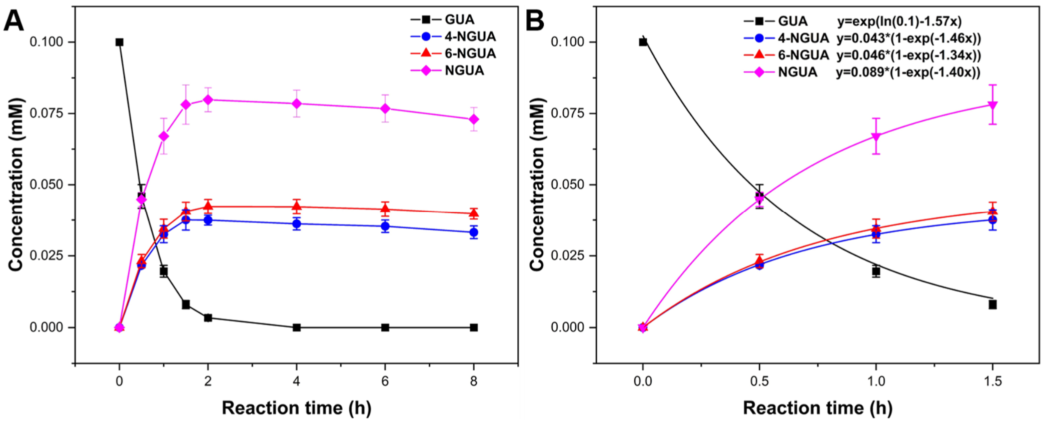

Figure S2. (A) Concentration profiles of GUA (guaiacol), 4-NGUA (4-nitrosoguaiacol), 6-NGUA (6-nitrosoguaiacol), and NGUA (4-nitrosoguaiacol + 6-nitrosoguaiacol) for 8 hours. (B) The fitting functions for the concentration changes of GUA, 4-NGUA, and 6-NGUA. Experimental conditions: [GUA] = 0.1 mM, [$NaNO_2$] = 1 mM, pH = 3.0 ± 0.1, in the dark, with zero air bubbling, at room temperature. The black, blue, red and pink lines represent GUA, 4-NGUA, 6-NGUA and NGUA, respectively.

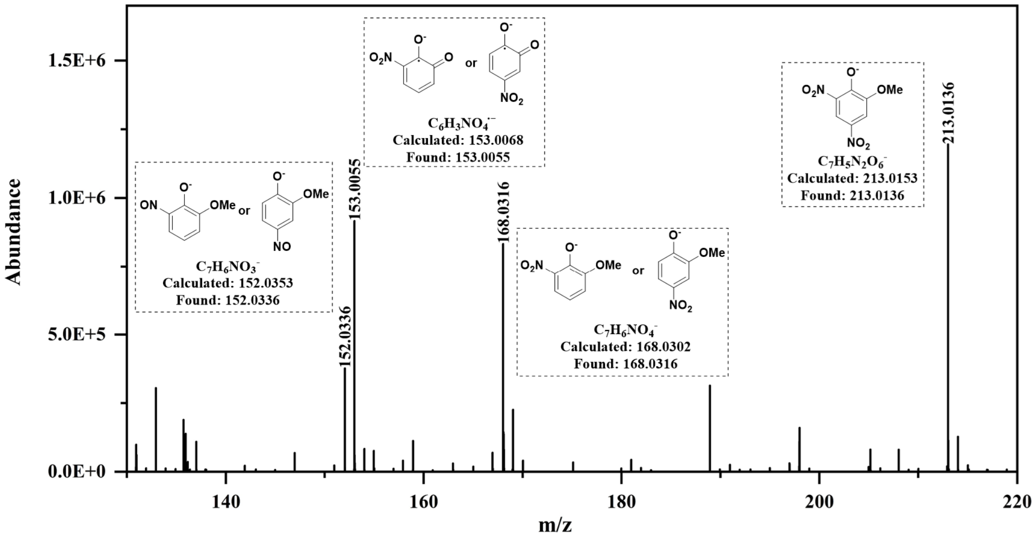

Figure S3. The HRMS of the reaction of GUA and $NaNO_2$ after 8h. Experimental conditions: [GUA] = 0.1 mM, [$NaNO_2$] = 1 mM, pH = 3.0 ± 0.1, without light, zero air bubbling, room temperature.

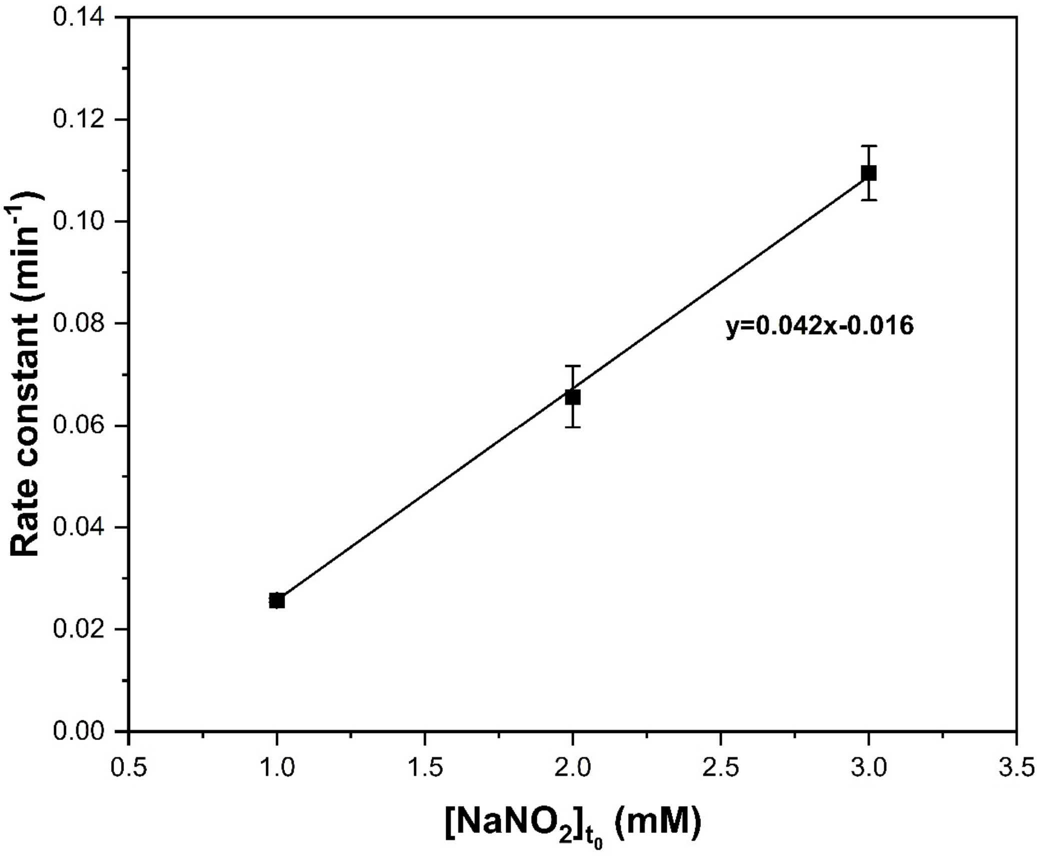

Figure S4. The dependence of the pseudo-first-order rate constant for the GUA + $NaNO_2$ reaction on the concentration of $NaNO_2$. Experimental conditions: [GUA] = 0.1 mM, pH = 3.0 ± 0.1, without light, zero air bubbling, room temperature.

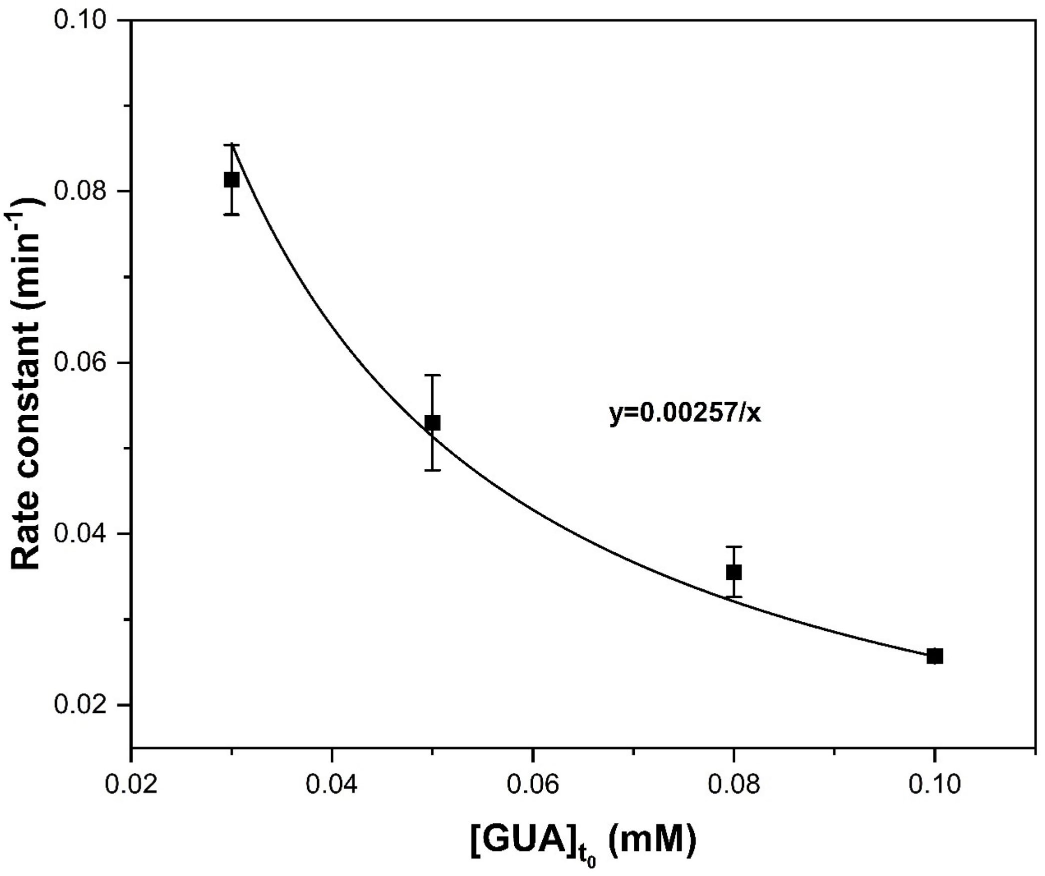

Figure S5. The dependence of the pseudo-first-order rate constant for the reaction of GUA with $NaNO_2$ on the concentration of GUA. Experimental conditions: [$NaNO_2$] = 1.0 mM, pH = 3.0 ± 0.1, without light, zero air bubbling, room temperature. GUA concentration

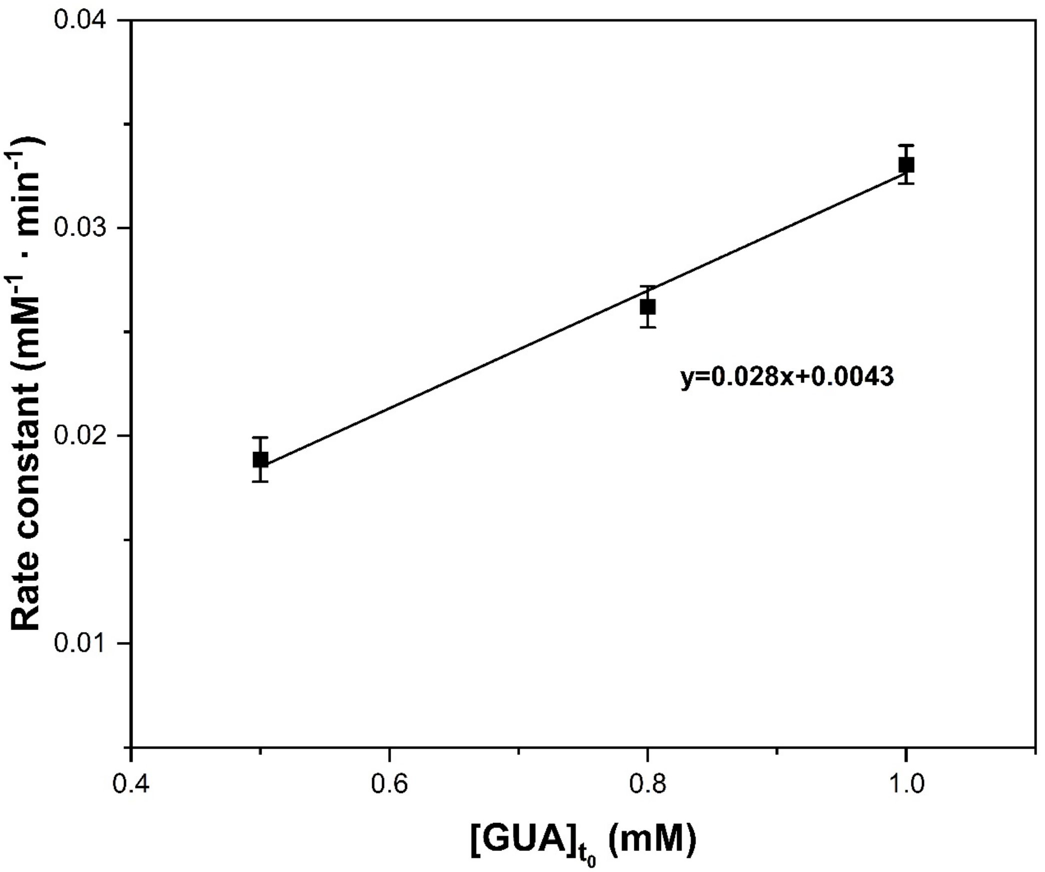

Figure S6. The dependence of the pseudo-second-order rate constant for the reaction of GUA with $NaNO_2$ on the concentration of GUA. Experimental conditions: $[NaNO_2]$ = 1.0 mM, pH = 3.0 ± 0.1, without light, with zero air bubbling, room temperature.

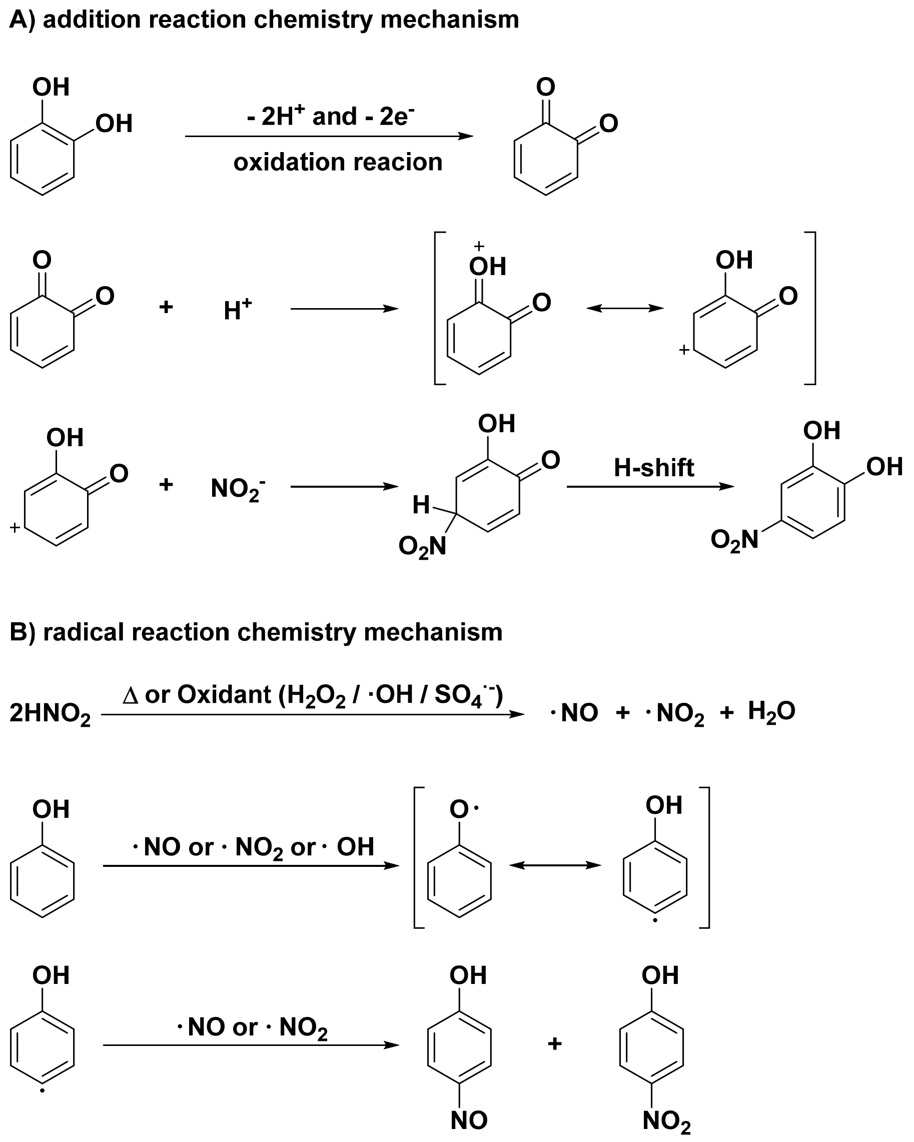

Figure S7. Two chemical mechanisms for the reactions between N(III) inorganic species and phenols. (A) Addition reaction pathway in solutions with a pH above 5.5 (6). (B) Radical reaction pathway in solutions with a pH below 5.5 (7).

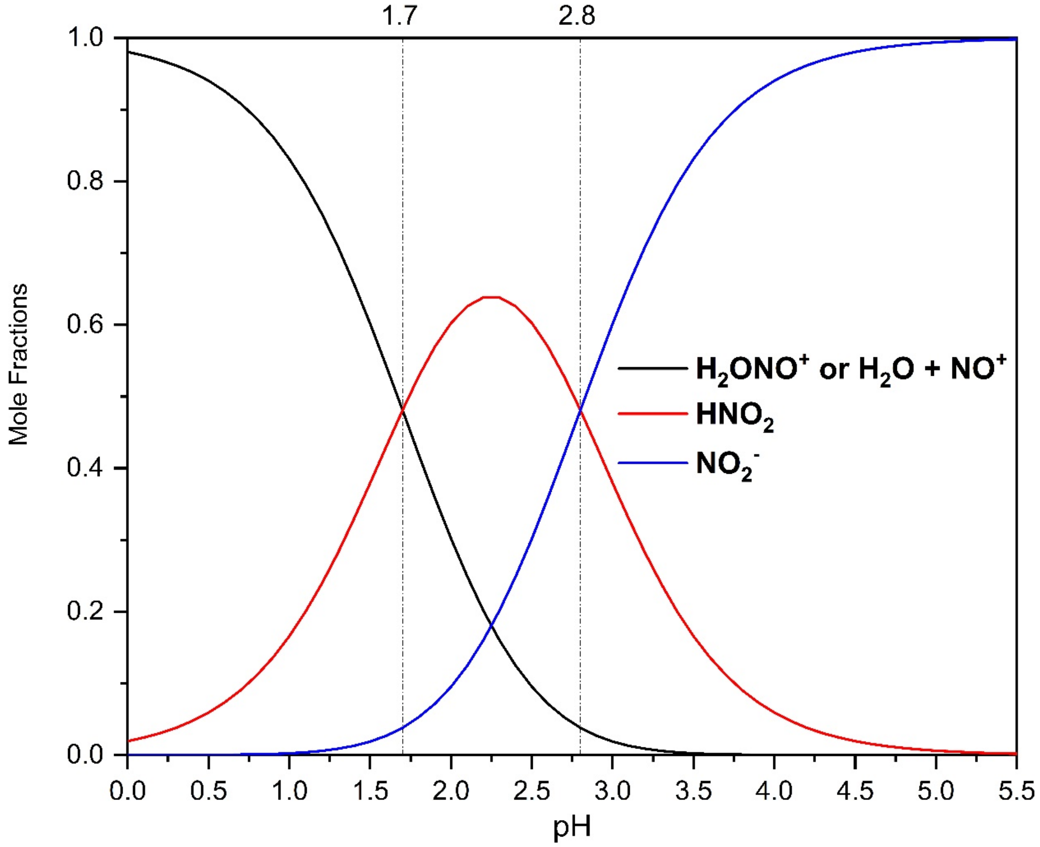

Figure S8. The ion speciation of N(III) inorganic compounds according to the p*K*a. In theory, when the pH level of the solution surpasses 3.5, the primary constituents are HNO_2 and NO_2^-. However, when the pH falls below 1.0, the primary constituents are H_2ONO^+ and HNO_2. The black, red and blue lines represent H_2ONO^+, HNO_2, and NO_2^-.

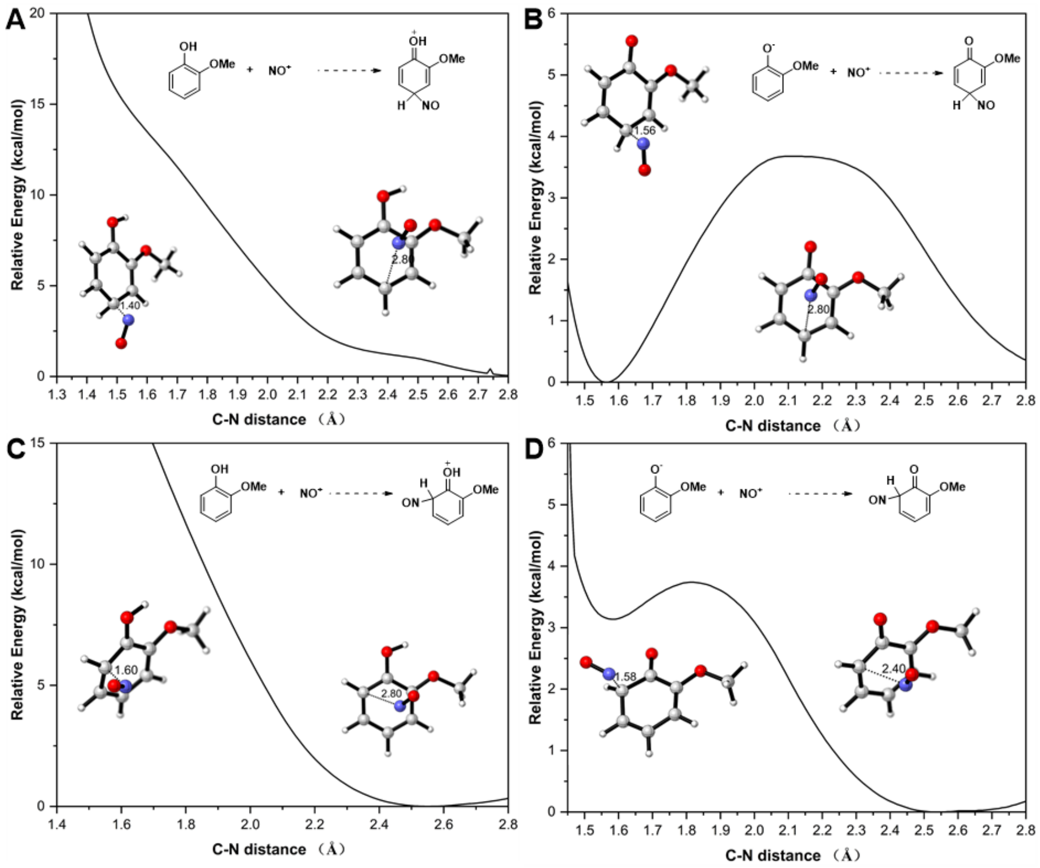

Figure S9. The modredundant calculation abbreviates the distance between the N atoms of NO+ and the C atoms of the benzene ring. (A) and (B) To form 4-nitrosoguaiacol from guaiacol and guaiacol$^-$. (C) and (D) To form 6-nitrosoguaiacol from guaiacol and guaiacol$^-$. The gray, white, red and blue balls represent C, H, O and N atoms, respectively.

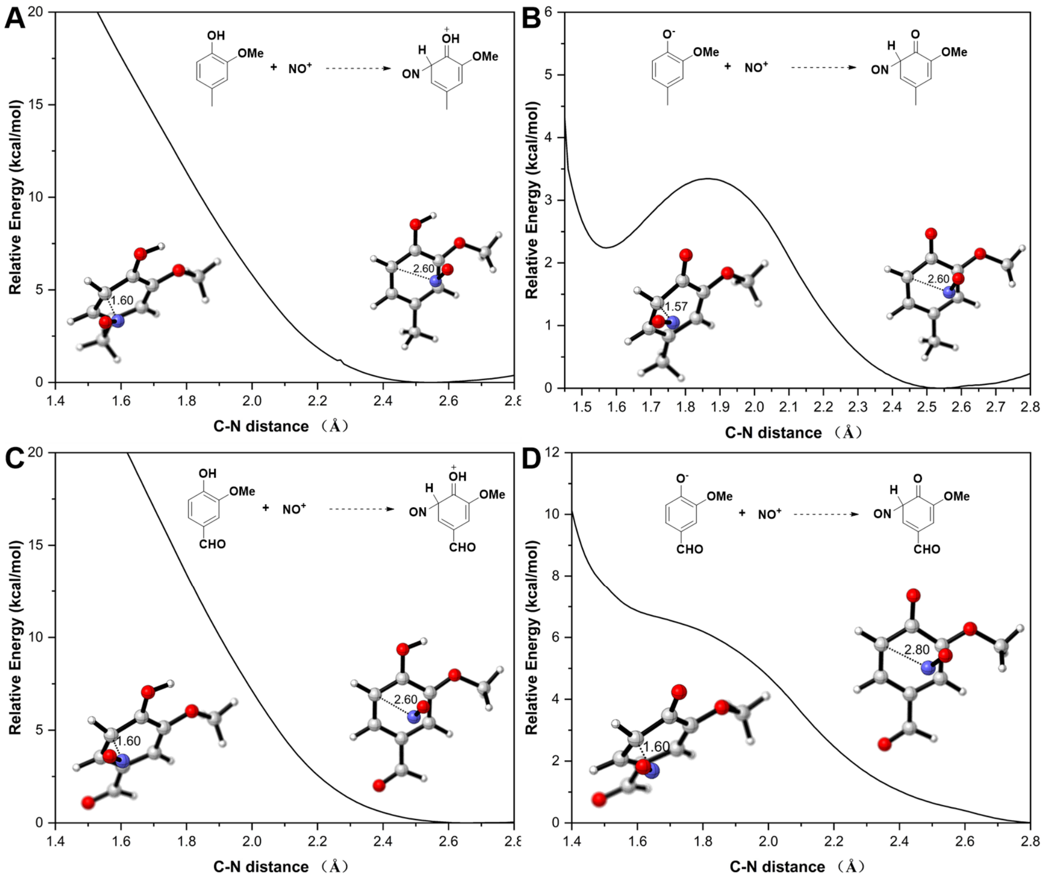

Figure S10. The modredundant calculation abbreviates the distance between the N atoms of NO^+ and the C atoms of the benzene ring. (A) and (B) for the formation of 6-nitrosocreosol from creosol and creosol$^-$. (C) and (D) for the formation of 6-nitrosovanillin from vanillin and vanillin$^-$. The gray, white, red and blue balls symbolize C, H, O, and N atoms, respectively.

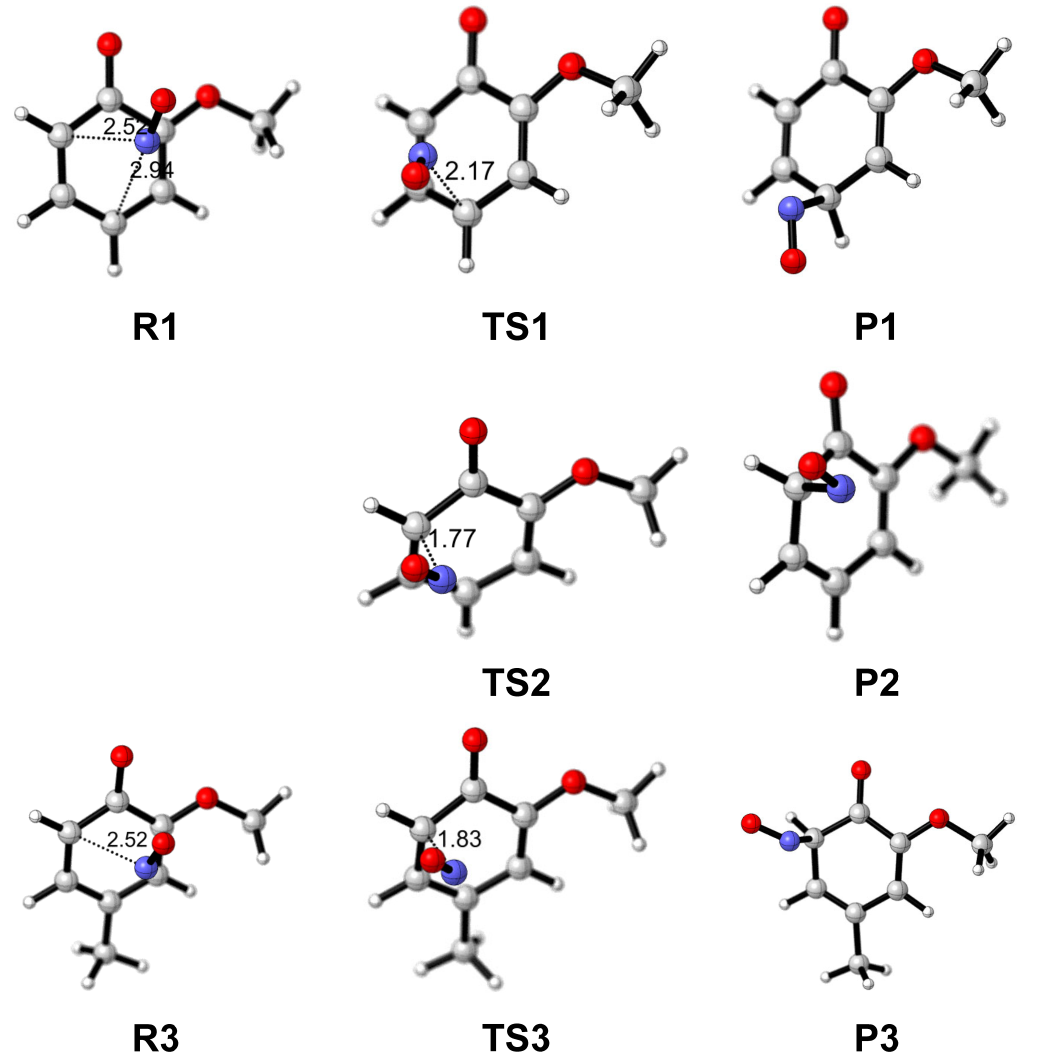

Figure S11. The molecular structures along the reaction coordinates cited (Å) in Figure 3A and 3B. The gray, white, red and blue balls symbolize C, H, O, and N atoms, respectively.

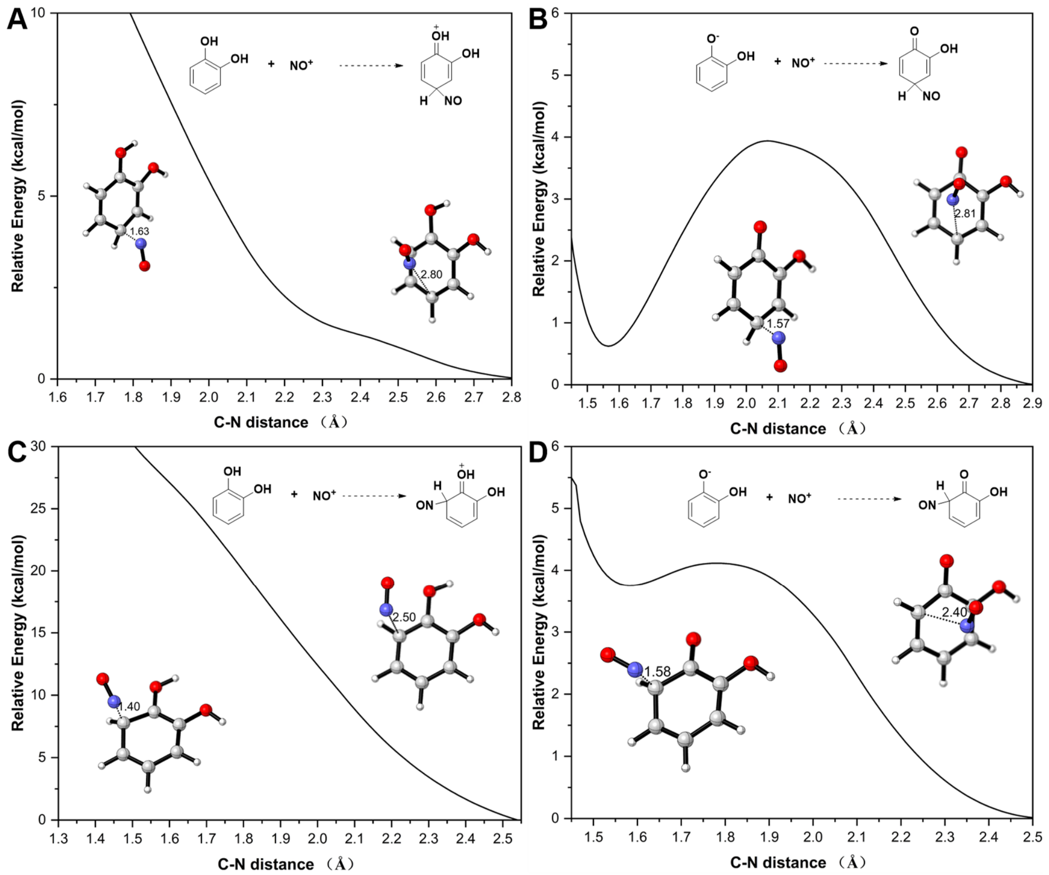

Figure S12. The modredundant calculation abbreviates the distance between the N atoms of NO^+ and the C atoms of the benzene ring. (A) and (B) To form 4-nitrosocatechol from catechol and catechol⁻. (C) and (D) To form 6-nitrosocatechol from catechol and catechol⁻. The gray, white, red and blue balls symbolize C, H, O, and N atoms, respectively.

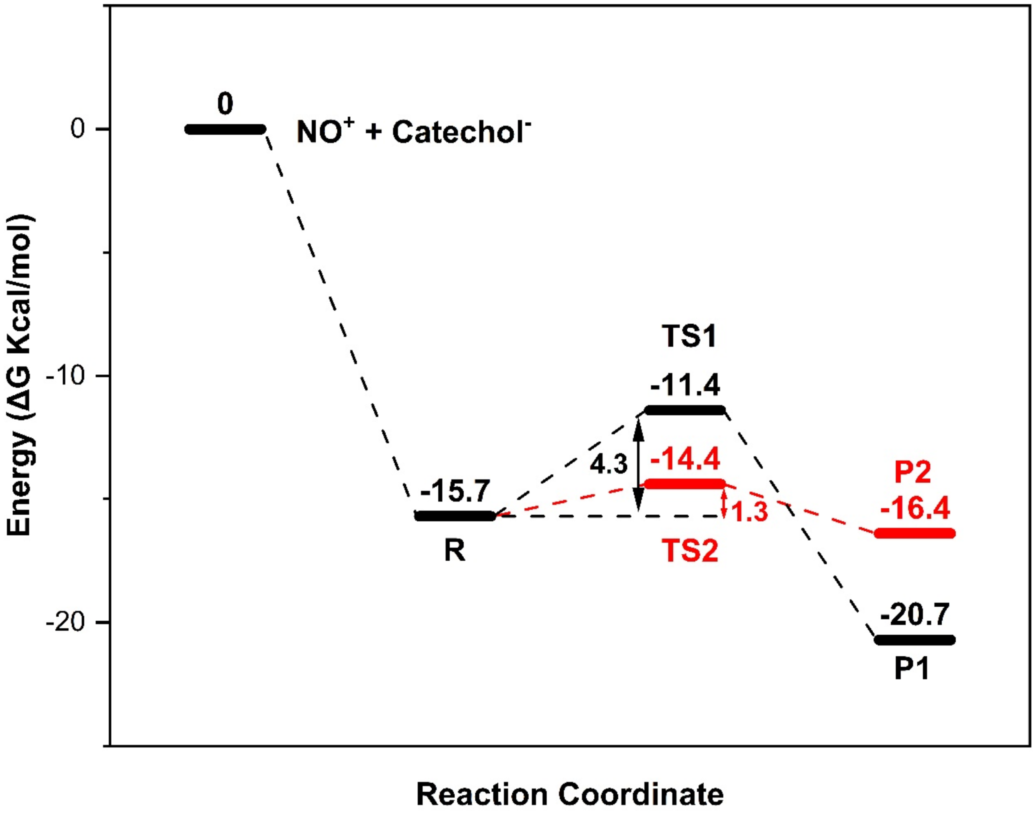

Figure S13. Gibbs free-energy (in kcal/mol at 298.15 K) profiles for the reaction of catechol and NO^+ at the DLPNO-CCSD(T)/aug-cc-pVTZ/SMD(water)//B3LYP-D3(BJ)/aug-cc-pVTZ/SMD(water) level of theory with the Zero Point Energy (ZPE) correction applied. The black line represents the reaction mechanisms for forming 4-nitrosocatechol and the red line represents the reaction mechanisms for forming 6-nitrosocatechol.

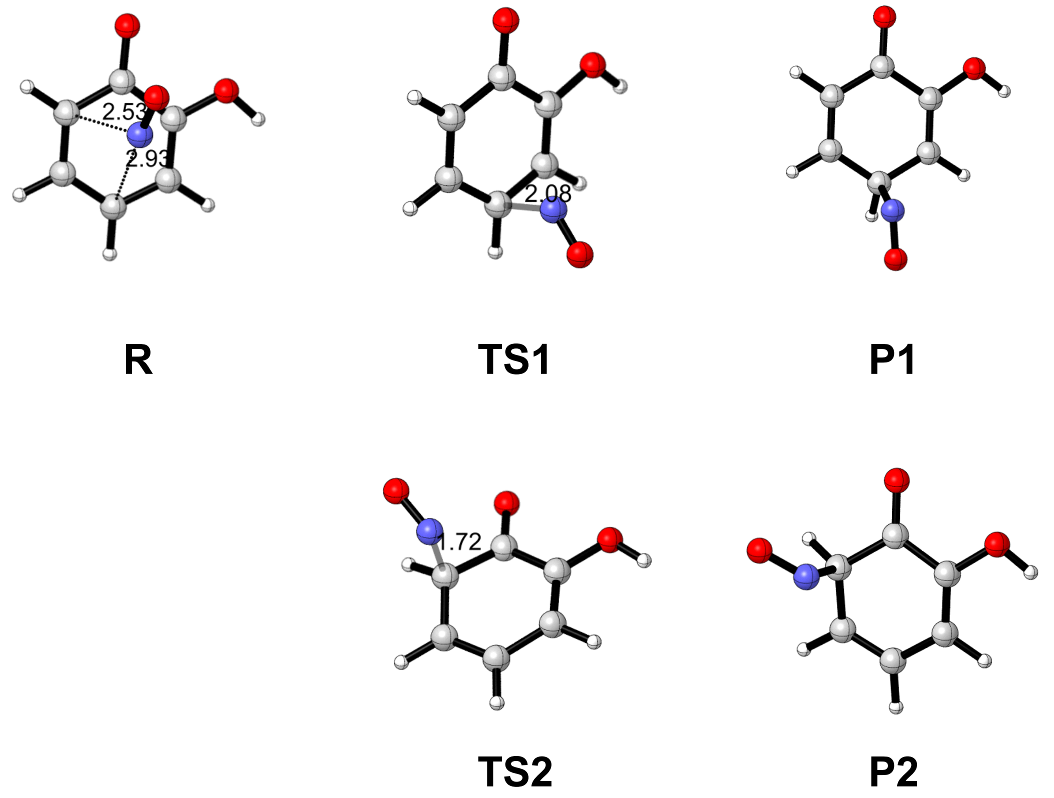

Figure S14. The molecular structures along the reaction coordinates cited (Å) in Figure S13. The gray, white, red and blue balls symbolize C, H, O, and N atoms, respectively.

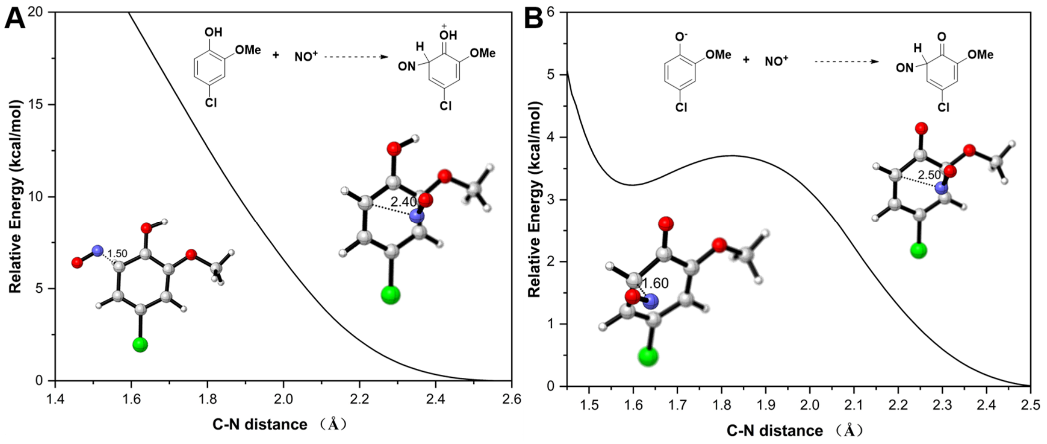

Figure S15. The modredundant calculation abbreviates the distance between the N atoms of NO^+ and the C atoms of the benzene ring. A) 4ClGUA. B) $4ClGUA^-$. The gray, white, red, blue and green balls symbolize C, H, O, N and Cl atoms, respectively.

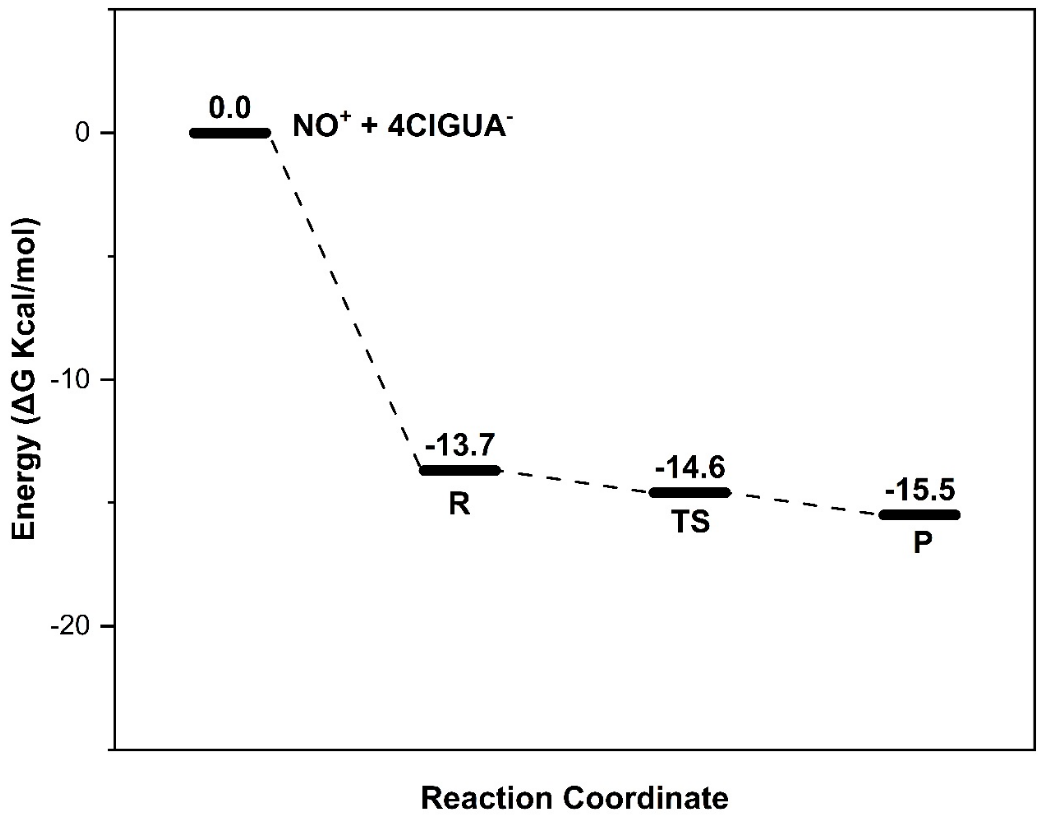

Figure S16. Gibbs free-energy (in kcal/mol at 298.15 K) profiles for the reaction of catechol and NO^+ at the DLPNO-CCSD(T)/aug-cc-pVTZ/SMD(water)//B3LYP-D3(BJ)/aug-cc-pVTZ/SMD(water) level of theory with the Zero Point Energy (ZPE) correction applied. The black line represents the reaction mechanisms for forming 4-chloro-2-methoxy-6-nitrosophenol.

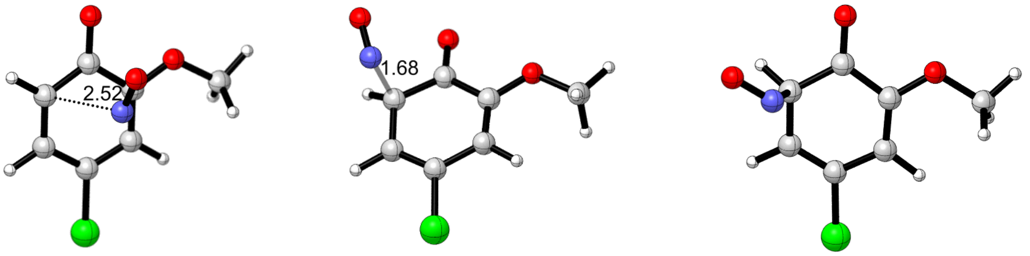

R **TS** **P**

Figure S17. The molecular structures along the reaction coordinates cited (Å) in Figure S16. The gray, white, red, blue and green balls symbolize C, H, O, N and Cl atoms, respectively.

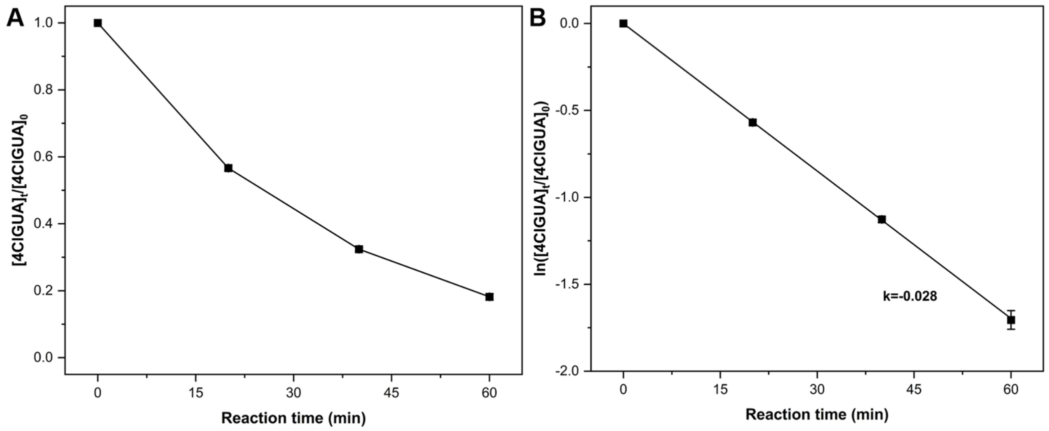

Figure S18. (A) The kinetics of the dark aqueous-phase reaction between 4ClGUA and $NaNO_2$. (B) The first-order rate constant for this reaction. Experimental conditions: [4ClGUA] = 0.1 mM, [$NaNO_2$] = 1 mM, pH = 3.0 ± 0.1, without light, with zero air bubbling, room temperature.

Table S1. The experiments conducted in this work. [a] The pseudo-first-order rate constant (min^{-1}). [b] The pseudo-second-order rate constant ($mM^{-1} \cdot min^{-1}$). [c] Bubble using zero air with 84 ppb O3.

entry	Organics	[Org] (mM)	[$NaNO_2$] (mM)	gas	pH	temperature	rate constant [a]	Half-life (min)
1	Guaiacol	0.1	1.0	Zero air	3.0	rt (22℃)	~0.026	~27
2	Guaiacol	0.1	2.0	Zero air	3.0	rt (22℃)	~0.066	~11
3	Guaiacol	0.1	3.0	Zero air	3.0	rt (22℃)	~0.109	~6
4	Guaiacol	0.03	1.0	Zero air	3.0	rt (22℃)	~0.081	~9
5	Guaiacol	0.05	1.0	Zero air	3.0	rt (22℃)	~0.053	~13
6	Guaiacol	0.08	1.0	Zero air	3.0	rt (22℃)	~0.036	~19
7	Guaiacol	0.5	1.0	Zero air	3.0	rt (22℃)	~0.018 [b]	~40
8	Guaiacol	0.8	1.0	Zero air	3.0	rt (22℃)	~0.025 [b]	~32
9	Guaiacol	1.0	1.0	Zero air	3.0	rt (22℃)	~0.033 [b]	~30
10	Creosol	0.1	1.0	Zero air	3.0	rt (22℃)	~0.104	~7
11	Syringol	0.1	1.0	Zero air	3.0	rt (22℃)	~0.162	~4
12	Eugenol	0.1	1.0	Zero air	3.0	rt (22℃)	~0.081	~9
13	Vanillin	0.1	1.0	Zero air	3.0	rt (22℃)	~0.001	~693
14	Vanillin-acid	0.1	1.0	Zero air	3.0	rt (22℃)	~0.003	~231
15	Syringaldehyde	0.1	1.0	Zero air	3.0	rt (22℃)	~0.004	~173
16	Guaiacol	0.1	1.0	Zero air	1.3	rt (22℃)	~0.052	~13
17	Guaiacol	0.1	1.0	Zero air	2.0	rt (22℃)	~0.172	~4
18	Guaiacol	0.1	1.0	Zero air	3.5	rt (22℃)	~0.012	~58
19	Guaiacol	0.1	1.0	Zero air	4.0	rt (22℃)	~0.001	~693
20	Guaiacol	0.1	1.0	Zero air	4.5	rt (22℃)	~0.0004	~1733
21	Guaiacol	0.1	1.0	Zero air	3.0	5℃	~0.0124	~56
22	Guaiacol	0.1	1.0	Zero air	3.0	35℃	~0.078	~9
23	Guaiacol	0.1	1.0	N_2	3.0	rt (22℃)	~0.014	~50

24 [c]	Guaiacol	0.1	1.0	84 ppb O_3	3.0	rt (22℃)	~0.023	~30
25	4ClGUA]	0.1	1.0	Zero air	3.0	rt (22℃)	~0.028	~25

S3. Model Calculation.

We calculated the concentrations of phenols in the gas and aqueous phases as a function of liquid water content at 5 °C. The Henry's law constants ($K_{H,\,278K}$) of guaiacol, catechol, syringol, m-benzenediol, and p-benzenediol at 278 k were calculated from measured K_H,289 K and the enthalpy of dissolution (ΔH_{sol}) (8) (see Table S2):

$$K_{H,278K} = K_{H,298K} \times \exp\left(\frac{\Delta H_{sol}}{R} \times \left(\frac{1}{298K} - \frac{1}{278K}\right)\right)$$

R is the ideal-gas constant 8.314 J K^{-1} mol^{-1}.

We calculate the distribution factor $f_{phenols}$ and the aqueous phase X_{aq} using:

$$f_{phenols} = 10^{-6} K_{H,279K} RTL = K_{H,279K} RT w_L$$

$$X_{aq} = \frac{f_{phenols}}{1 + f_{phenols}}$$

R is the ideal-gas constant 0.08205 atm L mol^{-1} K^{-1}. T is the temperature 278 K. L is the cloud/fog liquid water content in g m^{-3}.

We calculated the degradation rate of phenols with HONO and NO_3 radical in the aqueous phases at pH 3.0 and temperature 5 °C. The degradation rate of phenols with oxidants calculates using:

$$R_{ox} = k_{phenols+Ox} \times [phenols]_{aq} \times [Ox]_{aq}$$

In the aqueous-phase reaction between HONO and phenols, the active reactive species are phenols$^-$ and NO^+. Previous studies have reported the total reaction rate constants for phenols and NO^+. Therefore, the degradation rate of phenols is calculated using the total reaction rate constant as follows:

$$R_{HONO} = k_{phenols+NO^+} \times [phenols]_{aq} \times [NO^+]_{aq}$$

$k_{phenols+NO^+}$ is 2.2×10^9 M^{-1} s^{-1} for phenol (9). Our experiments show that with the increase of electron cloud density on the aromatic ring, the degradation rate will increase by 3-6 times. Therefore, we estimate that the second-order reaction rate constant $k_{guaiacol+NO^+}$ of guaiacol and NO^+ is 1.32×10^{10} M^{-1} s^{-1}, and the $k_{\text{syringol}+NO^+}$ is 7.9×10^{10} M^{-1} s^{-1}. The concentrations of HONO, NO^+ in the aqueous phase are given by (10, 11):

$$[HONO]_{aq} = [HONO]_g K_{H,HONO,pH=3} \quad K_{H,HONO,pH=3} = 46\ M\ atm^{-1}$$

$$\textbf{HONO + H}^+ \longrightarrow \textbf{NO}_2^+ \textbf{ + H}_2\textbf{O} \quad \textbf{K}_a \textbf{ = 2×10}^{-7}\ \textbf{M}^{-1}\ \textbf{(20 °C)}$$

$$[NO^+]_{aq} = K_a [HONO]_{aq} [H^+]$$

So that:

$$R_{HONO} = k_{phenols+NO^+} \times [phenols]_{aq} \times K_a [H^+][HONO]_g K_{H,HONO,pH=3}$$

The degradation rate of phenols with NO_3 radical calculates using:

$$R_{NO_3} = k_{phenols+NO_3} \times [phenols]_{aq} \times [NO_3]_{aq}$$

$k_{phenols+NO_3}$ is 5×10^7 M^{-1} s^{-1} for guaiacol. There is no available $k_{phenols+NO_3}$ value for syringol, we assume it has the same value as guaiacol (12). $[NO_3]_{aq}$ is 1×10^{-12} M, represents a typical nighttime cloud droplet concentration (13).

Therefore, the contribution of phenolic compounds to the degradation rate at nighttime aqueous phase can be calculated using:

$$\frac{R_{HONO}}{R_{NO_3\,radical}} = \frac{k_{phenols+NO^+} \times [phenols]_{aq} \times K_a[H^+][HONO]_g K_{H,HONO,pH=3}}{k_{phenols+NO_3} \times [phenols]_{aq} \times [NO_3]_{aq}}$$

$$\frac{R_{HONO}}{R_{NO_3\,radical}} = \frac{k_{phenols+NO^+} \times K_a[H^+][HONO]_g K_{H,HONO,pH=3}}{k_{phenols+NO_3} \times [NO_3]_{aq}}$$

Table S2. The Henry's law constants of guaiacol, catechol, syringol, m-benzenediol, and p-benzenediol (8).

phenol	$K_{H,298K}$ (M atm^{-1})	$K_{H,278K}$ (M atm^{-1})
guaiacol	870	5326
catechol	5000	25199
syringol	4700	21523
m-benzenediol	8.4×10^{6}	3.8×10^{7}
p-benzenediol	2.3×10^{7}	1.0×10^{8}

SI References

1. A. D. Becke, Density-functional thermochemistry. III. The role of exact exchange. *J. Chem. Phys.* **98**, 5648-5652 (1993).
2. S. Grimme, S. Ehrlich, L. Goerigk, Effect of the damping function in dispersion corrected density functional theory. *J. Comput. Chem.* **32**, 1456-1465 (2011).
3. A. V. Marenich, C. J. Cramer, D. G. Truhlar, Universal Solvation Model Based on Solute Electron Density and on a Continuum Model of the Solvent Defined by the Bulk Dielectric Constant and Atomic Surface Tensions. *J. Phys. Chem. B* **113**, 6378-6396 (2009).
4. M. Frisch *et al.*, Gaussian 16, Revision A. 03, Gaussian. *Inc., Wallingford CT* **3** (2016).
5. C. Y. Legault (2009) CYLview, 1.0b. in http://www.cylview.org (Université de Sherbrooke).
6. K. Vidović, A. Kroflič, P. Jovanovič, M. Šala, I. Grgić, Electrochemistry as a Tool for Studies of Complex Reaction Mechanisms: The Case of the Atmospheric Aqueous-Phase Aging of Catechols. *Environ. Sci. Technol.* **53**, 11195-11203 (2019).
7. A. Kroflič, M. Huš, M. Grilc, I. Grgić, Underappreciated and Complex Role of Nitrous Acid in Aromatic Nitration under Mild Environmental Conditions: The Case of Activated Methoxyphenols. *Environ. Sci. Technol.* **52**, 13756-13765 (2018).
8. A. S. McFall, A. W. Johnson, C. Anastasio, Air–Water Partitioning of Biomass-Burning Phenols and the Effects of Temperature and Salinity. *Environ. Sci. Technol.* **54**, 3823-3830 (2020).
9. S. González-Mancebo, M. P. García-Santos, J. Hernández-Benito, E. Calle, J. Casado, Nitrosation of Phenolic Compounds: Inhibition and Enhancement. *J. Agric. Food Chem.* **47**, 2235-2240 (1999).
10. T. A. Turney, G. A. Wright, Nitrous Acid And Nitrosation. *Chem. Rev.* **59**, 497-513 (1959).
11. Y. Wang, S. Jorga, J. Abbatt, Nitration of Phenols by Reaction with Aqueous Nitrite: A Pathway for the Formation of Atmospheric Brown Carbon. *ACS Earth Space Chem.* **7**, 632-641 (2023).
12. P. Barzaghi, H. Herrmann, Kinetics and mechanisms of reactions of the nitrate radical (NO3) with substituted phenols in aqueous solution. *Physical Chemistry Chemical Physics* **6** (2004).
13. W. L. Chameides, Possible role of NO_3 in the nighttime chemistry of a cloud. *J. Geophys. Res. Atmos.* **91**, 5331-5337 (1986).
